# Supplementary material for: Establishing and augmenting views on the acceptability of a paediatric critical care randomised controlled trial (the FEVER trial): a mixed methods study
Source: BMJ Open. 2021 Mar 10;11(3):e041952. doi: 10.1136/bmjopen-2020-041952 (PMC7949453; doi:10.1136/bmjopen-2020-041952)
Supplement: Author's manuscript [file bmjopen-2020-041952.draft_revisions.pdf]

BMJ Open

BMJ Open is committed to open peer review. As part of this commitment we make the peer review history of every article we publish publicly available.

When an article is published we post the peer reviewers' comments and the authors' responses online. We also post the versions of the paper that were used during peer review. These are the versions that the peer review comments apply to.

The versions of the paper that follow are the versions that were submitted during the peer review process. They are not the versions of record or the final published versions. They should not be cited or distributed as the published version of this manuscript.

BMJ Open is an open access journal and the full, final, typeset and author-corrected version of record of the manuscript is available on our site with no access controls, subscription charges or pay-per-view fees (http://bmjopen.bmj.com).

If you have any questions on BMJ Open's open peer review process please email info.bmjopen@bmj.com

BMJ Open

Establishing and augmenting acceptability of a paediatric critical care trial: a mixed methods study

Journal:	*BMJ Open*
Manuscript ID	bmjopen-2020-041952
Article Type:	Original research
Date Submitted by the Author:	21-Jun-2020
Complete List of Authors:	Deja, Elizabeth; University of Liverpool, Department of Public Health, Policy and Systems<br>Peters, Mark; Great Ormond Street Hospital For Children NHS Trust, Paediatric Intensive Care Unit; University College London, Institute of Child Health<br>Khan, Imran ; Queen Mary University of London, Institute of Population Health Sciences<br>Mouncey, Paul; ICNARC, Clinical Trials Unit<br>Agbeko, Rachel; Great North Children's Hospital, Paediatric Intensive Care Unit<br>Fenn, Blaise; Patient partner<br>Watkins, Jason; Patient partner<br>Ramnarayan , Padmanabhan; Great Ormond Street Hospital for Children, Children's Acute Transport Service,<br>Tibby, Shane; Evelina Childrens Hospital, Paediatric Intensive Care<br>Thorburn, Kentigern; Alder Hey ChilDr.ens Hospital, Paediatric Intensive Care<br>Tume, Lyvonne; University of Salford, School of Health and Society<br>Rowan, Kathryn; Intensive Care National Audit and Research Centre (ICNARC),<br>Woolfall, Kerry; University of Liverpool,
Keywords:	Paediatric intensive & critical care < INTENSIVE & CRITICAL CARE, PAEDIATRICS, PAIN MANAGEMENT, QUALITATIVE RESEARCH

SCHOLARONE™
Manuscripts

I, the Submitting Author has the right to grant and does grant on behalf of all authors of the Work (as defined in the below author licence), an exclusive licence and/or a non-exclusive licence for contributions from authors who are: i) UK Crown employees; ii) where BMJ has agreed a CC-BY licence shall apply, and/or iii) in accordance with the terms applicable for US Federal Government officers or employees acting as part of their official duties; on a worldwide, perpetual, irrevocable, royalty-free basis to BMJ Publishing Group Ltd ("BMJ") its licensees and where the relevant Journal is co-owned by BMJ to the co-owners of the Journal, to publish the Work in this journal and any other BMJ products and to exploit all rights, as set out in our licence.

The Submitting Author accepts and understands that any supply made under these terms is made by BMJ to the Submitting Author unless you are acting as an employee on behalf of your employer or a postgraduate student of an affiliated institution which is paying any applicable article publishing charge ("APC") for Open Access articles. Where the Submitting Author wishes to make the Work available on an Open Access basis (and intends to pay the relevant APC), the terms of reuse of such Open Access shall be governed by a Creative Commons licence – details of these licences and which Creative Commons licence will apply to this Work are set out in our licence referred to above.

Other than as permitted in any relevant BMJ Author's Self Archiving Policies, I confirm this Work has not been accepted for publication elsewhere, is not being considered for publication elsewhere and does not duplicate material already published. I confirm all authors consent to publication of this Work and authorise the granting of this licence.

For peer review only

Establishing and augmenting acceptability of a paediatric critical care trial: a mixed methods study

Elizabeth Deja[1] PhD, Mark J Peters PhD [2], Imran Khan MSc[3] Paul R Mouncey MSc,[3] Rachel Agbeko FFICM,[4] Blaise Fenn,[5] Jason Watkins,[5] Padmanabhan Ramnarayan FFICM,[6] Shane M Tibby MB.ChB,[7] Kentigern Thorburn MMed MD,[8] Lyvonne N Tume PhD,[9] Kathryn M Rowan PhD [3] and, Kerry Woolfall PhD[1] on behalf of the Paediatric Intensive Care Society Study Group (PICS-SG)

Affiliations:

1 Institute of Population Health Sciences, University of Liverpool, Liverpool, United Kingdom
2 Respiratory, Critical Care and Anaesthesia Unit, University College London, Great Ormond Street, Institute of Child Health, London, United Kingdom
3 Institute of Population Health Sciences, Queen Mary University of London, United Kingdom
4 Paediatric Intensive Care Unit, Great North Children's Hospital, The Newcastle upon Tyne Hospitals NHS Foundation Trust, Institute of Cellular Medicine, Newcastle University, Newcastle, United Kingdom
5 Patient partner
6 Children's Acute Transport Service, Great Ormond Street Hospital, London, UK
7 Guy's & St Thomas' NHS Foundation Trust, Evelina Children's Hospital
8 Paediatric Intensive Care Unit, Alder Hey Children's Hospital, Liverpool, UK
9 Faculty of Health and Applied Sciences, University of the West of England, Glenside Campus, Bristol, United Kingdom

Address correspondence to:

Dr Elizabeth Deja. University of Liverpool, 1st Floor, Block B, Waterhouse Building, Liverpool, L69 3GL. Email: E.Deja@liverpool.ac.uk. Telephone: 0151 794 5613

Word count: 3500

For peer review only

For peer review only - http://bmjopen.bmj.com/site/about/guidelines.xhtml

ABSTRACT

Objective: To explore how research with parents and staff established and augmented perspectives and the design of a randomised controlled trial investigating temperature thresholds in critically ill children with fever and infection (FEVER).

Design: Mixed methods feasibility study at three time points: 1) before, 2) during and 3) after a pilot trial.

Setting: English, Paediatric Intensive Care Unities (PICU).

Participants: 1) pre-pilot trial focus groups with pilot site staff (n=56) and interviews with parents(n=25) whose child had been admitted to PICU in the last three years with a Fever and suspected infection; 2) questionnaires with parents of randomised children following pilot trial recruitment (n=48 from 47 families) 3) post-pilot trial interviews with parents (n=19), focus groups (n=50) and a survey (n=48) with site staff. Analysis drew on Sekhon et al's (2017) theoretical framework of acceptability.

Results: There was initial support for the trial, yet some concerns regarding proposed temperature thresholds and not using paracetamol for pain or discomfort. Pre-trial findings informed protocol changes and training. Some concerns about pain or discomfort during weaning from ventilation remained. Staff trained by the FEVER team found the trial more acceptable than those trained by colleagues. Parents and staff found the trial acceptable.

Conclusions: Pre-trial findings and pilot trial experience influenced acceptability, providing insight into how challenges may be overcome. We present an adapted theoretical framework of acceptability to inform future trial feasibility studies.

KEY WORDS

Acceptability, Clinical trial, feasibility, Paediatric intensive care, qualitative, sepsis.

ARTICLE SUMMARY

Strengths and limitations of this study

- The longitudinal design enabled collection of data from parents and staff with relevant experience before, during and after the pilot trial.
- The mixed methods approach including interviews, focus groups and questionnaires enabled breadth and depth of insight to help establish trial feasibility.
- Use of the Sekhon, Cartwright and Francis (2017) Theoretical Framework of Acceptability allowed trial acceptability to be evaluated as a multifaceted construct as opposed to a poorly defined binary (acceptable/not acceptable) approach.
- Data collected during the pilot trial stage were limited to parent perspectives, the majority of whom were mothers, although staff views were sought retrospectively.

For peer review only

INTRODUCTION

Recruitment and retention in clinical trials is a significant challenge, which leads to underpowered trials and the continued use of healthcare interventions that are not informed by robust scientific evidence [1-3]. Paediatric clinical trials are particularly challenging to conduct due to ethical and practical considerations that are not applicable to trials in adult settings [4-7]. For example, the eligible population is smaller and consent is obtained by proxy through children's parents or legal guardians [4, 8, 9]. These considerations are compounded in critical care settings by the emotive and time sensitive situation in which they take place. Clinical trials must be acceptable to parents and health care practitioners to facilitate recruitment, adherence and consent [10, 11]. Sekhon, Cartwright and Francis (2017)[11] present a Theoretical Framework of Acceptability (TFA) (see Figure 1) to assist researchers in assessing the acceptability of healthcare interventions, including clinical trials. The TFA presents seven theoretical constructs for researchers to consider when assessing whether people delivering or receiving a healthcare intervention consider it to be appropriate. The constructs highlight considerations when establishing acceptability, such as how an individual feels about the healthcare intervention, the perceived burden of taking part and the extent to which the participant understands the healthcare intervention and how it works. To our knowledge, the TFA has not been used in the analysis of real world data.

[INSERT FIGURE 1: Sekhon, Cartwright and Francis (2017) Theoretical Framework of Acceptability]

There is a lack of robust evidence or guidance to inform the management of fever due to an infection in critically ill children. The FEVER feasibility study aimed to establish whether it is possible to conduct a clinical trial comparing a permissive approach to fever (treat at $\geq 40^{o}$C) with a restrictive approach (treat at $\geq 37.5^{o}$C). Perceived challenges to the successful conduct of a fever randomised control trial (RCT) included: a protocol that was likely to differ from usual clinical practice; potential parental and staff concerns about allowing a child's fever to rise without treatment; no time to seek informed consent [12, 13]; and the possibility that children may die before trial participation is discussed with parents.

The FEVER feasibility study involved pre-trial research with parents and staff, an observation of UK practice and a subsequent pilot RCT with embedded research exploring the perspectives of parents and staff involved in the pilot RCT. Pilot RCT and observation study findings are reported separately [14, 15]. This paper focuses on research conducted to explore parent and staff perspectives on trial acceptability though the lens of the TFA.

For peer review only - http://bmjopen.bmj.com/site/about/guidelines.xhtml

METHODS

Study design

Mixed methods research involving interviews, focus groups and questionnaires during the pre- trial and pilot RCT. We used previous research [16, 17] to develop topic guides and participant information sheets (PIS). Exploring parent and staff views on the acceptability of FEVER, including information materials, temperature thresholds and the use of research without prior consent (RWPC).

Patent and public involvement

Two parents (CF and JW) and one young adult (BF) with experience of severe infection and admission to hospital were co-investigators and members of the SMG. They provided valuable input into the design and conduct of the study, including reviewing documents for parent interviews (e.g. draft pilot trial participant information sheets) and informing study recruitment approaches (i.e. identification of social media groups and charities). They were also involved in the review of study progress and findings**.**

Pre pilot trial: prospective recruitment and conduct

Parent interviews

Parents of children that had been admitted to an intensive care unit with a fever and infection in the last three years were recruited via: a database from a previous relevant study [18]; a letter from study sites [15]; and adverting on relevant social media and at sites. All routes invited parents to register interest in participation by contacting the research team.

Psychologist ED (PhD, female research associate) responded to parents' requests to participate in sequential order and checked eligibility. A draft pilot RCT PIS was emailed to parents prior to interview, which took place with ED in person or via telephone based on parent preference, consent was obtained. Interviewing stopped when data saturation [19] and variation in sample was reached.

Staff focus groups

Co-investigators at the four pilot RCT sites [15] disseminated invitations to relevant staff. KW (PhD, female, social scientist, Senior Lecturer) or ED obtained written consent. Closed questions were administered using a voting system (Turning Technologies, Youngstown, OH, USA).

For peer review only - http://bmjopen.bmj.com/site/about/guidelines.xhtml

Pilot RCT: Concurrent and retrospective recruitment and conduct

Interim analysis of prospective data informed subsequent topic guides and questionnaires.

Parent questionnaire and interviews

As part of the pilot RCT consent discussions, site researchers asked parents if they would like to complete the FEVER consent questionnaire before hospital discharge (concurrent) and/or take part in a telephone interview approximately a month later (retrospective). ED contacted those who consented to interview in sequential order, stratifying by study arm (lower/higher temperature threshold).

Staff survey and focus groups

At the end of the pilot RCT, ED repeated focus groups with staff. Those unable to attend a focus group were invited by email to complete an online questionnaire containing the same closed questions administered to focus group participants using the voting system.

Analysis

Digital audio recordings were transcribed verbatim by a professional transcription company (Voicescript Ltd., Bristol, UK). Transcripts were anonymised and checked for accuracy.

ED and KW used a thematic analysis approach [20] to explore themes within the data related to views on trial design and acceptability. Analysis was interpretive and iterative [20, 21] . NVivo 10 software (QSR International Pty Ltd., Melbourne, Australia) was used to assist in the organisation and coding of qualitative data. Quantitative data from the parent and staff questionnaires were entered into SPSS Version 20.0 (IBM Corp., Armonk, NY, US) and analysed using chi-squared and descriptive statistics. Please see separate publication for further details [15]. ED and KW then synthesized data and used framework analysis [22] to map findings onto each component of the TFA by time point [11] (see tables 1-3).

For peer review only - http://bmjopen.bmj.com/site/about/guidelines.xhtml

Table 1: Prospective Acceptability of a FEVER Pilot Trial mapped to Sekhon et al's acceptability framework (2017)

Group & data collection method	Affective attitude	Burden	Ethicality	Intervention Coherence	Opportunity Costs	Perceived Effectiveness	Self-Efficacy
**Parents**<br><br>Interviews	100% stated they would consent for their child to take part in a Fever RCT<br><br>Would consent with a 40°C threshold, but 39.5-39.9°C more acceptable<br>*"I think 39.5 [°C]. But again you guys know best I'm just saying …that's very hot.* (P17, father, non-bereaved)	The intervention was not invasive<br><br>Concerns about unnecessary discomfort /pain in higher threshold	Belief it is important to help other children in the future.<br><br>Use of RWPC is necessary *"'I understand there's not really another way you can do it"* (P01, mother, non-bereaved)	Logically 'make sense' (P02, mother, bereaved).<br>PIS "it's simple for them to read. (P06, mother, bereaved)<br><br>Many suggested changes to the PIS to assist understanding and decision making	Children would still be given all other kinds of care/ interventions<br><br>Concerns about loss of non-antipyretics effects of paracetamol e.g. reducing risk of seizures/riggers, pain relief.	When study rationale was explained parents understood how allowing a fever could have a positive impact: *"Fever is meant to be like part of a fighting off, healing process isn't it?"* (P07, mother, non-bereaved)	The intervention was something parents understood and said they could support Important to approach for RWPC when parents have the capacity to make an informed decision
**Staff**<br><br>Focus group	82% indicated 39.5°C was an acceptable permissive temperature threshold.<br>18.2% suggested 40°C was acceptable. Only 20.4% suggested 37.5°C was acceptable as may lead to unnecessary intervention.	Watching a child be in pain or experience negative side effects: *"Incredibly difficult to wait and watch"* (P05, FG5). The trial would be more acceptable if limited to ventilated children.	Mixed views on RWPC, n=25/49, (51%) thought acceptable based on past experience and the emergency situation.<br><br>Concerns about use of RWPC for an intervention that may not be supported by parents.	Understanding that optimal temperature thresholds are unknown<br><br>Want more clinical evidence as it goes against experiential knowledge (e.g. administering antipyretic at 38°C)	Concerns about the loss of non-antipyretic effects e.g. discomfort relief, reducing risk of seizures/riggers, decreased cardio work load.<br><br>Staff with no experience of RWPC had concerns it would negatively impact on trust and the "*working relationship*" (P03,FG1)	Evidence to support the trial*: "Well there is, there is a bit of science which suggests we should let the temperature get higher"* (P01, FG3)<br><br>Waiting for the permissive threshold would go against their clinical training or "*gut instinct*" (P05, FG2)	No perceived issues with taking a temperature. Query method that is going to be used.<br><br>Nurses stated they may not follow the protocol if a child was upset, combative, in discomfort

For peer review only - http://bmjopen.bmj.com/site/about/guidelines.xhtml

Table 2: Patent concordant acceptability of a FEVER Pilot Trial mapped to Sekhon et al's acceptability framework (2017)

Data collection method	Affective attitude	Burden	Ethicality	Intervention Coherence	Opportunity Costs	Perceived Effectiveness	Self-Efficacy
**Parents**<br><br>Questionnaire	N= 92/100 (92%) consented to taking part in the Fever Pilot RCT.	Not collected at this Time point	N= 32/41 (79%) reported the belief medical research studies are important<br><br>N= 42/48 (89.4%) satisfied with the RWPC process in the Fever pilot RCT<br><br>N= 32/41 (78%) selected helping other children as a reason for taking part.	N= 46/48, (96%) agreed that the information received about the Fever pilot RCT was clear & straightforward to understand	*My child is comfortable*" (P49, questionnaire, mother, permissive)<br><br>Concerns about their child being in pain or discomfort and impact on pre-existing medical condition.<br>"*My son had too many underlying medical conditions and felt it may hinder his recovery as he was selected to the upper limit before treatment* (P73, questionnaire, father, permissive)	N= 30/41 (73.2%) selected helping my child as a reason for taking part.<br><br>"*So far recovering well*" P21,mother,non-bereaved)	N= 41,(89.4%) felt they made the decision for their child to take part in the pilot trial

For peer review only - http://bmjopen.bmj.com/site/about/guidelines.xhtml

Table 3: Respective Acceptability of a FEVER Pilot Trial mapped to Sekhon et al's acceptability framework (2017)

**Group & data collection method(s)**	**Affective attitude**	**Burden**	**Ethicality**	**Intervention Coherence**	**Opportunity Costs**	**Perceived Effectiveness**	**Self-Efficacy**
**Parents**<br>Interviews	*'I think it's a brilliant idea, so I'm all, I'm all for it"* (P80, interview mother, permissive).<br>Decliners also approve the trial *"They seem to be quick let's give them paracetamol. we've always been like a kind of, a hold off [. . .] I think sometimes paracetamol can hide other things going on as well."* (P83, interview, mother, permissive, decliner)	Not invasive *" I mean if it was more of an invasive study, I might have had to query it a little bit more but I was happy with everything"* (Parent 53, interview, father, restrictive).<br><br>Not in discomfort while ventilated	Not causing harm to their child: *"I just thought, There's no harm in letting her into the study"* (P78, interview, mother, restrictive).<br><br>Wish to help others "if it's gonna help other people then yeah "(P85, interview, father, restrictive).<br><br>RWPC was acceptable	Involved treatment, which parents were familiar with; that logically made sense; that was *"Clearly explained"* (P74, interview, mother, permissive),	37.5°C ,very acceptable as normal practice at home<br><br>39.5°C acceptable if child is not in discomfort	Understand how a fever may logically help a child.	Valued ability to withdraw or decline consent. "*I was happy enough for him to undergo the trial but if at any point the nurses thought he could do with the Calpol, or I thought he could, then I wanted the trial to stop it could do.*" (P49, interview, mother, permissive)
		When being weaned of venerator some children displayed discomfort / distress.			≥39.5 °C not acceptable if the child is in pain or distress.	Concerns about negative impact due to child's pre-existing medical conditions	
**Staff**<br><br>Focus group & Survey	85 % (n=81/95) trial acceptable<br><br>95% (n=95/100, four missing) 37. °C acceptable<br><br>53% (n=42/79) 39.5°C acceptable	If the child is not conscious or not in pain no burden	Parents think the trial is acceptable therefore it is ethical to randomise their children.<br><br>89.4 % (n= 42/48) satisfied with the use of RWPC	If trained by trial team. Then 39.5°C acceptable	Mixed views, some observed no negative side effects and reported no costs.	Seeing as believing, *"It's a really good, valuable study to see on a larger scale"* (P04, FG5).	Valued being involved in trial design. Ability to follow the protocol mixed, n= 52/96 (54%) *"technically very easy to follow"*(*P06,* FG1)

For peer review only - http://bmjopen.bmj.com/site/about/guidelines.xhtml

RESULTS

Participant characteristics

Prospective (*Pre-pilot RCT*)*:* 25 semi-structured interviews (n=20 mothers, n=5 fathers) with bereaved (n=6) and non-bereaved (n=19) parents (see figure 2). Parents were interviewed a median of 14 months (range 6-38 months) after admission. Interviews took a median of 48 minutes (range 15-105 minutes). The 15-minute interview was concluded part way though by a bereaved father. Fifty-six staff took part in six focus groups across the four sites, lasting a median of 50 minutes (range 31-59 minutes). Staff mainly self-identified as nurses (n=45, 81%), all were involved in the clinical care of children.

[INSERT FIGURE 2: Participant characteristics by time point]

Concurrent (*During-pilot RCT*)*:* Eighty parents of the 100 children randomised to the pilot RCT consented to receive questionnaire, of these, 48 from 47 families completed and returned a questionnaire while their child was admitted to hospital. Of these, 41/48 (85%) provided consent and 6/48 (13%) declined consent (n=1 missing).

Retrospective (*Post-pilot RCT*)*:* Sixty- six parents of the 100 children randomised to the FEVER pilot trial consented to be contacted for an interview. Data saturation [19] was reached after eight interviews with parents of children allocated to the restrictive (lower) temperature threshold and after 11 interviews with parents of children allocated to the permissive (higher) temperature threshold. Parents were interviewed a median of 31 days after randomisation (range 9-70 days). Their children had received treatment for respiratory illness (e.g. bronchiolitis and respiratory syncytial virus) (n=18/19, 94%), cancer (n=1/19, 5%) and septic shock (n=1/19, 5%). Interviews took an average 32 minutes (range 20-50 minutes).

The staff sample included 98 site staff across all four pilot RCT sites. Almost half (48/98, 49 %) completed the questionnaire, with the rest attending a focus group. The majority (n=75, 77%) were nurses, n=45 (60%) were senior-level staff and most (n=79/98, 81%) were involved in the clinical care of children. Focus groups took an average of 53 minutes (range 23-106 minutes).

Pre-trial, prospective acceptability

All parents interviewed described how they would hypothetically consent for the use of their child's information in the proposed trial. Parents' views on trial acceptability appeared to be influenced by factors including: all other treatments for infection are given (*Opportunity costs*); the non-invasive nature of the intervention (*Burden*); support for RWPC in this context (*Ethicality*)*;* trust in medical

For peer review only - http://bmjopen.bmj.com/site/about/guidelines.xhtml

staff to act in the best interests of their child; and a belief that the trial question made sense and therefore likely to achieve its purpose (*Perceived effectiveness*):

> *"'cause fever is meant to be like part of a fighting off, healing process isn't it? A natural one… I can understand exactly why it would be interesting to see what happens."* (*P07, mother, non-bereaved*)

Although analysis of pre-RCT data indicated that many of the constructs of acceptability were met (see Table 1), there were also aspects of burden, opportunity costs, ethicality and intervention coherence identified as problematic by both parents and staff. As shown in Table 1, staff concerns outweighed support at this stage.

The majority of parents were not worried about the proposed restrictive temperature threshold of 37.5°C. However, staff expressed concerns that this was too low a threshold to administer an antipyretic (n=43/54, 80%, two missing) and would go against perceived "*normal practice*" (P01, FG4). A common concern was that children would be given unnecessary treatments in a clinical context where "*we try and give the minimum amount of drugs*" (P02, FG5) (*Affective attitude*).

In contrast, many parents voiced concerns about the acceptability of the permissive threshold with regards to increased risk of "*seizures*" (P03, mother, non-bereaved) and other potential detrimental side effects (opportunity costs), such as "*organs shutting down"* (P07, mother, non-bereaved), "*rigger*"" (P06, mother, bereaved) or unnecessary discomfort (*Burden*). The majority suggested that the pilot RCT would be more acceptable if the permissive temperature threshold was slightly lower (e.g. 39.9°C or 39.5°C). Although parents stated that they would still consent to a trial involving a threshold of 40°C (*Affective attitude*), as they trusted staff to monitor their child and act in their best interests:

> "*I would trust that my child was being monitored, it's not like they're waiting for her condition to get worse before they do something, you are having, a nurse by your bedside at all times, I had complete trust.*" (P25, mother, non-bereaved)

Staff also described how a permissive threshold of ≥40°C was too high and how they would be concerned about not using paracetamol for analgesia in the less unwell, spontaneously breathing patients, who may be in pain or discomfort (*Burden*). In addition, staff were concerned about parental acceptability of the permissive threshold, RWPC (*Ethicality, Opportunity costs*) and the impact of increased cardiac workload (*Opportunity costs*).

Both groups understood the aims of the proposed trial. However, in addition to changes to temperature thresholds, amendments to the protocol were suggested. Parents identified aspects of

For peer review only - http://bmjopen.bmj.com/site/about/guidelines.xhtml

the PIS that required clarification, including whether not treating a temperature could cause a seizure, incorporating an explanation of how all other treatments would still be given. Staff requested additional information about the scientific evidence underpinning the research question, as well as clarification on key issues, such as what cooling methods could be used.

Response to pre-trial findings

Pre-trial findings were used in conjunction with observation study findings [15] to develop the pilot RCT protocol and site training. These included: a permissive temperature threshold of ≥39.5°C; inclusion criteria that required patients to be mechanically ventilated, therefore likely to be on other analgesia and changes to information materials. For example, staff training and PIS incorporated evidence to demonstrate how fever does not cause seizures and observation study findings that showed the restrictive temperature threshold (≥37.5°C) falls within usual practice. To address staff concerns about how parents may respond to trial and RWPC discussions, parent perspectives were communicated in site training, highlighting parental acceptability of RWPC, temperature thresholds, parents' questions about the study, and suggestions on how to address such questions.

Concurrent acceptability

As demonstrated in Table 2, parent questionnaire data showed that the six constructs of acceptability measured during this time point were met. Parents reported that the study information '*was clear and straightforward to understand'* (*Intervention coherence*). Ninety two percent of randomised children received consent for their continued participation in the pilot RCT (Affective attitude) [15]. Main reasons for providing consent related to the belief that participation might help their child (n=30/41, 73%, *Perceived effectiveness*) and help other children in the future (n=32/41, 78%, *Ethicality*). Parents also found the study acceptable because "*my child was comfortable*" (P49, questionnaire, mother, permissive) (*Opportunity costs and Burden*).

Of the eight that refused consent to continue, seven (88%) had been allocated to the permissive (higher) treatment group. Data suggested that parents who declined some element of their child participation still supported the proposed Fever RCT. Reasons for refusal of consent were linked to pre-existing medical conditions and the wish to limit any discomfort experienced by their child: *"My son had too many underlying medical conditions and felt it may hinder his recovery as he was selected to the upper limit"* (P73, questionnaire, father, permissive). Suggesting that there were still some concerns about withholding analgesia (*Opportunity costs*).

Staff acceptability was not measured at this point.

For peer review only - http://bmjopen.bmj.com/site/about/guidelines.xhtml

Post-trial, retrospective acceptability

Interviews conducted with parents a median one-month post randomisation supported and provided further insights into questionnaire findings. All seven constructs of acceptability were met (see table 3). Parents were interested in the trial question and felt the proposed trial was important (*Affective attitude, Perceived effectiveness*). Parents described staff as approaching them appropriately, with well-timed, clear, comprehensive study information leading to strong intervention coherence:

> *"I understood what they were saying and was happy to sort of go ahead, with the trial. If it wasn't explained to me too well, I probably wouldn't have bothered doing it."* (P77, interview, father, restrictive) (*Intervention coherence*)

Parents of children allocated to the restrictive temperature threshold found the trial very acceptable as giving paracetamol at this temperature was "*something that I would do myself anyway"* (P82, interview, father, restrictive) (*Ethicality*). Parents also viewed the permissive threshold to be acceptable. However, this acceptability was conditional on their child not being in discomfort (*Opportunity costs*):

> *"The only thing would be if she wasn't on any other kind of pain relief, but there's other things to manage, her discomfort".* (P73, interview, father, permissive)

Indeed, two mothers described how they found the trial acceptable and gave full consent, but later chose to withdraw their child from the study when they were being weaned from ventilation and sedation due to concerns about their child being in pain or distress. Parents valued the ability to withdraw or decline consent (*Self-efficacy*). They also described how they trusted staff to act in their child's best interests, including not adhering to the protocol by administering an antipyretic if at any point staff felt that it was needed (*Burden and Opportunity costs*):

> *"I know if anything did happen, you's can stop at any time. Stop it if they saw it was getting out of hand and he, and I felt like it, it wasn't helping, that I would stop it….they wouldn't let him go to the stage of him getting poorly"*. (P85, interview, father, restrictive)

Unlike parents' views, which largely remained consistent across study time points, staff perceptions of the acceptability of the lower temperature shifted during the course of the pilot RCT. Witnessing patient's positive reactions to RWPC and trial discussions and an awareness that ≥37.5°C was usual practice, resulted in 95% (n=95/100, four missing) of staff rating the restrictive threshold as acceptable or very acceptable: "*Everybody that was in the lower end of it, I found were like happy to take part*" (P01, FG4).

For peer review only - http://bmjopen.bmj.com/site/about/guidelines.xhtml

Staff had mixed views about the acceptability of the permissive temperature threshold. Approximately half (n=42/79, 53%) indicated that the ≥39.5°C threshold was acceptable. They valued how the trial team responded to their pre-trial concerns by changing the inclusion criteria to omit non-ventilated children (*Self-efficacy*). Some stated that their previous concerns about high temperatures causing harm or discomfort (*Opportunity costs*) and parents having a negative response to the trial and RWPC (*Ethicality*) were not observed:

> *"Some patients are randomised into the higher temperature and people see that they're actually manageable and it doesn't cause them any harm… It's kind of seeing is believing."* (P03, FG1)

Staff who did not find the permissive temperature acceptable were concerned about not giving paracetamol for pain or discomfort when a child was conscious (*Opportunity costs*). These concerns meant that some staff administered paracetamol before a child's temperature had reached ≥39.5 °C: *"I feel like potentially we're making our patients more uncomfortable."* (P01, FG2)

Interestingly, staff trained by their local unit colleagues were significantly more likely to find the permissive threshold not acceptable when compared to those trained directly by the pilot trial team ($X^2(2)$ =8.78, p = 0.012). Staff trained colleagues also rated site training as being poor (n=11/97, 11%). These staff remained unclear about the scientific rationale for the study and had lower Intervention coherence.

Despite issues with aspects of *Intervention coherence* and *Opportunity costs*, overall staff rated the fever trial acceptable (n=81/95, 85 %, *Affective attitude*) and practicable to conduct (n=80/95, 84%, *Self-efficacy*). Findings suggest that their views could be further augmented if the proposed Fever RCT protocol was revised to also exclude patients receiving non-invasive forms of ventilation (e.g. high-flow nasal oxygen) or those close to being extubated when sedation is being weaned.

Trust

During data analysis, we found that the concept of trust between parents and staff was prevalent within our data and intransigently linked to trial acceptability. For example, parents found the trial acceptable because they trusted staff to put the needs of their child before the requirements of the study. Both groups discussed the trust parents place in medical expertise during a very emotive situation. Staff also highlighted that maintaining parental trust impacts on their decisions "*I feel like there's an element of trust there that would be broken from my point of view*." (P01, FG2,

For peer review only - http://bmjopen.bmj.com/site/about/guidelines.xhtml

retrospective). The construct of 'Trust' is not reflected within the TFA. We present an adapted TFA in Figure 3 incorporating Trust.

[INSERT FIGURE 3: The adapted Theoretical framework of Acceptability)

DISCUSSION

Our study highlights the value of conducting pre-trial research with all key stakeholders to inform the design of challenging clinical trials [23]. Research with parents and staff, helped establish trial acceptability, as well as influence and changed perspectives over time. Prospective qualitative research identified mixed staff views, whilst parents found the trial broadly acceptable. Both the parental and staff support for RWPC in time critical trials is constant with previous research [18, 24-28]. Aspects of *Intervention Coherence, Opportunity Costs, Ethicality and Burden* [11] were identified that threatened trial success. The majority of staff concerns related to not using paracetamol or active cooling for pain relief, or to prevent febrile seizures [14]. Prospective findings informed changes to the PIS, staff training package and the addition of mechanical ventilation to inclusion criteria. Data from the concurrent and retrospective time points showed a positive response to such changes, particularly amongst staff. Suggestions to further augment views on trial acceptability and reduce the number of potential protocol deviations and withdrawals were identified. These include changes to trial inclusion criteria as well as staff training content and delivery [14].

Our findings demonstrate Sekhon and Francis' (2017) assertion that the acceptability of healthcare interventions is not a fixed construct. If we had taken a binary (acceptable/ not acceptable) or snapshot approach to determining acceptability, then we would not have been able to identify and address key concerns that threatened trial acceptability and ultimately, trial feasibility. The TFA was demonstrated to be comprehensive and relevant to our work. However, we found that the concept of trust between parents and staff was closely linked to trial acceptability and is not reflected in the framework. The importance of trust is a recurring theme in healthcare and medicine but is particularly salient in paediatric trials, as the more vulnerable the population, the greater the need for trust [5, 29]. Drawing on Hall et al's (2001) [29] work into defining trust in medical relationships, we propose the addition of an eighth construct of 'Trust' to help inform future trial feasibility research (see Figure 3). Further research is needed to test the adapted model in establishing the feasibility of other healthcare interventions and settings. This work will help to establish the appropriateness of trust as additional construct in the TFA.

As the pilot trial was conducted in three months, during the busy winter period the concurrent work only included parents and therefore lacks insight into staff perspectives during pilot trial conduct.

For peer review only - http://bmjopen.bmj.com/site/about/guidelines.xhtml

This limitation was compensated for by the use of retrospective (1 week-1 month) mixed methods ensuring a larger sample, through the survey and depth of insights, through focus groups.

In summary challenges to delivering the proposed trial included staff and parent concerns about the acceptability of the proposed protocol. Pre-trial research, staff training and experience of pilot trial conduct augmented views, providing insight into how challenges may be overcome, such as changes to the inclusion criteria and delivery of site training. We present an adapted TFA to inform the design of future trial feasibility studies.

Acknowledgements

The authors would like to thank all the parents who shared their experiences with us; their contribution to the research is invaluable. The authors are grateful to all the staff at participating UK hospitals and charities/ support groups for their help with recruitment and the UK PICS Study Group (PICS-SG) for their contributions to this study. We would also like to thank Ms Clara Francis (CF) as co-investigator and patient partner.

Funding

This study was funded by the UK National Institute for Health Research Health Technology Assessment programme (15/44/01) and supported by the National Institute for Health Research Great Ormond Street Hospital Biomedical Research Centre. The trial sponsor was Great Ormond Street Hospital NHS Foundation Trust, Joint R&D Office GOSH/ICH, 30 Guilford Street, London WC1N 1EH, United Kingdom (email: Research.Governance@gosh.nhs.uk). The Clinical Trials Unit for the Fever study was the ICNARC CTU. The views expressed in this publication are those of the authors and not necessarily of the funder, the sponsor, the NHS, the NIHR, or the UK Department of Health.

Ethics approval and consent to participate

Ethical approval for the study was provided by the National Research Ethics Committee (NRES) (17/LO/1139). Management approvals were obtained from all study sites.

Data statement

The datasets generated during and/or analysed during the current study are not publicly available as consent was not sought for data sharing.

Competing interests' statement.

MJP is a member of the NIHR HTA Board. KMR is a member of the NIHR HS & DR Board.

For peer review only

Author contributions

Dr Elizabeth Deja conducted the qualitative research, co-analysed the data, drafted the initial manuscript, reviewed and revised the manuscript.

Professor Mark J Peters is the chief investigator of the FEVER study, he conceived, designed and oversaw conduct of the FEVER study and critically reviewed the manuscript.

Mr Imran Khan was the study manager at the ICNARC CTU responsible for day to day FEVER study management and critically reviewed the manuscript.

Mr Paul R Mouncey is the head of research at ICNARC CTU, a co-applicant, involved in the design and coordination of the FEVER study, contributed to and reviewed the manuscript.

Mr Blaise Fenn and Mr Jason Watkins were study co-applicants, patient and parent representatives who contributed to the design and conduct of the FEVER study including mixed methods perspectives elements.

Dr Padmanabhan Ramnarayan co-applicant helped design and conduct the FEVER study.

Dr Rachel Agbeko, Dr Shane M Tibby, Professor Lyvonne N Tume were co-applicants, site principal investigators and critically reviewed the manuscript.

Dr Kentigern Thorburn site principal investigator and critically reviewed the manuscript.

Dr Kathryn M Rowan is the director at ICNARC CTU, study co-applicant involved in designing and overseeing the FEVER study.

Dr Kerry Woolfall was a co-applicant on the FEVER study, designed the mixed methods perspectives elements of the study, supervised the qualitative research, co- analysed the data and reviewed and revised the manuscript.

Licence statement

I, the Submitting Author has the right to grant and does grant on behalf of all authors of the Work (as defined in the below author licence), an exclusive licence and/or a non-exclusive licence for contributions from authors who are: i) UK Crown employees; ii) where BMJ has agreed a CC-BY licence shall apply, and/or iii) in accordance with the terms applicable for US Federal Government officers or employees acting as part of their official duties; on a worldwide, perpetual, irrevocable, royalty-free basis to BMJ Publishing Group Ltd ("BMJ") its licensees and where the relevant Journal is co-owned by BMJ to the co-owners of the Journal, to publish the Work in BMJ Open and any other BMJ products and to exploit all rights, as set out in our licence.

REFRENCES

1. McDonald AM, Knight RC, Campbell MK, et al. What influences recruitment to randomised controlled trials? A review of trials funded by two UK funding agencies. . Trials;. 2006;7.

2. Bower P, Brueton V, Gamble C, et al. Interventions to improve recruitment and retention in clinical trials: a survey and workshop to assess current practice and future priorities. Trials. 2014;15(399).

3. Treweek S, Mitchell E, Pitkethly M, et al. Strategies to improve recruitment to randomised controlled trials. Cochrane Database Syst Re. 2010.

4. Caldwell P, Murphy S, Butow P, Craig J. Clinical trials in children The Lancet. 2004;364(9436):803-11.

For peer review only - http://bmjopen.bmj.com/site/about/guidelines.xhtml

5. Greenberg RG, Gamel B, Bloom D, et al. Parents' perceived obstacles to pediatric clinical trial participation: Findingsfrom the clinical trials transformation initiative Contemporary Clinical Trials Communications. 2018.
6. Sammons HM, Starkey ES. Ethical issues of clinical trials in children. Paediatrics and child health 2011;22(2).
7. Field MJ, Behrman RE. Institute of Medicine (U.S.). Committee on Clinical Research Involving Children., Ethical Conduct of Clinical Research Involving Children. Washington, D.C.: National Academies Press; 2004.
8. Caldwell PHY, Butow PN, Craig JC. Pediatricians' attitudes towards randomized controlled trials involving children. The Journal of Pediatrics 2002;141(6).
9. Caldwell P, Butow P, Craig J. Parents' attitudes to randomised controlled trials involving children. Journal of Pediatricts 2003;145:555–60.
10. Diepeveen S, Ling T, Suhrcke M, Roland M, Marteau TM. Public acceptability of government intervention to change health-related behaviours: a systematic review and narrative synthesis. BMC Public health 2013;13(756).
11. Sekhon M, Cartwright M, Francis JJ. Acceptability of healthcare interventions: an overview of reviews and development of a theoretical framework. BMC Health Services Research. 2017;17(88).
12. Proposal for a Regulation of the European Parliament and of the Council on Clinical trials on medicinal products for human use, and repealing Directive 2001/20/EC., (2013).
13. The Medicines for human use (Clinical trials) and Blood Safety and Quality (Amendment) Regulations., (2008).
14. Peters MJ, Woolfall K, Khan I, et al. Permissive versus restrictive temperature thresholds in critically ill children with fever and infection: a multicentre randomized clinical pilot trial. Critical Care. 2019;23(1):69.
15. Peters MJ, Woolfall K, Khan I, et al. Different temperature thresholds for antipyretic intervention in critically ill children with fever due to infection: the FEVER feasibility RCT. Health Technology Assessment 2019;23(5):1 - 148.
16. Woolfall K, Frith L, Gamble C, et al. How parents and practitioners experience research without prior consent (deferred consent) for emergency research involving children with life threatening conditions: a mixed method study. BMJ Open. 2015;5.
17. Inwald DP, Canter R, Woolfall K, et al. Restricted fluid bolus volume in early septic shock: results of the Fluids in Shock pilot trial. Arch Dis Child. 2018.
18. Inwald D, Canter R, Woolfall K, et al. Restricted fluid bolus versus current practice in children with septic shock: the FiSh feasibility study and pilot RCT. Health Technol Assess. 2018;22(51).
19. Saunder B, Sim J, Kingstone T, et al. Saturation in qualitative research: exploring its conceptualization and operationalization. Qual Quant. 2018;52(4).
20. Braun V, Clarke V. Using thematic analysis in psychology. Qualitative research in psychology. 2006;3(2):77-101.
21. Braun V, Clarke V. what can "thematic analysis’’offer health and wellbeing researchers? International journal of qualitative studies on health and well-being. 2014;9.
22. Gale NK, Heath G, Cameron E, Rashid S, Redwood S. Using the framework method for the analysis of qualitative data in multi-disciplinary health research. BMC Medical Research Methodology. 2013;13(117).
23. O'Cathain A, Thomas KJ, Drabble SJ, Rudolph A, Goode J, Hewison J. Maximising the value of combining qualitative research and randomised controlled trials in health research: the QUAlitative Research in Trials (QUART) study--a mixed methods study. Health Technol Assess. 2014;18(38):1-197, v-vi.
24. Canvin K, Jacoby A. Duty, desire or indifference? A qualitative study of patient decisions about recruitment to an epilepsy treatment trial. Trials. 2006;7(32).
25. Roper L, Sherratt FC, Young B, et al. Children’s views on research without prior consent in emergency situations: a UK qualitative study. BMJ open 2018;8.

For peer review only

For peer review only - http://bmjopen.bmj.com/site/about/guidelines.xhtml

26. Waterfield T, Lyttle MD, Shields M, et al. Parents' and clinicians' views on conducting paediatric diagnostic test accuracy studies without prior informed consent: qualitative insight from the Petechiae in Children study (PiC). Archives of Disease in Childhood. 2019:archdischild-2019-317117.
27. Woolfall K, Frith L, Dawson A, Gamble C, Lyttle MD, Young B. Fifteen-minute consultation: an evidence-based approach to research without prior consent (deferred consent) in neonatal and paediatric critical care trials. Archives of disease in childhood - Education & practice edition. 2016;101(1):49-53.
28. Brierley J, Larcher V. Emergency research in children: options for ethical recruitment. Journal of Medical Ethics. 2011;37(7):429-32.

29. Hall MA, Dugan E, Zheng B, Mishra AK. Trust in Physicians and Medical Institutions:What Is It, Can It Be Measured,and Does It Matter? The Milbank Quarterly. 2001;79(4).

For peer review only

For peer review only - http://bmjopen.bmj.com/site/about/guidelines.xhtml

LEDGENDS

Figure 1: Sekhon, Cartwright and Francis (2017) Theoretical Framework of Acceptability

Figure 2: Participant characteristics by time point

Table 1: Prospective Acceptability of a FEVER Pilot Trial mapped to Sekhon et al's acceptability framework (2017)

Table 2: Patent concordant acceptability of a FEVER Pilot Trial mapped to Sekhon et al's acceptability framework (2017)

Table 3: Respective Acceptability of a FEVER Pilot Trial mapped to Sekhon et als's acceptability framework (2017)

Figure 3: The adapted Theoretical framework of Acceptability

For peer review only

Figure 1, Theoretical Framework of Acceptability (Sekhon et al., 2017)

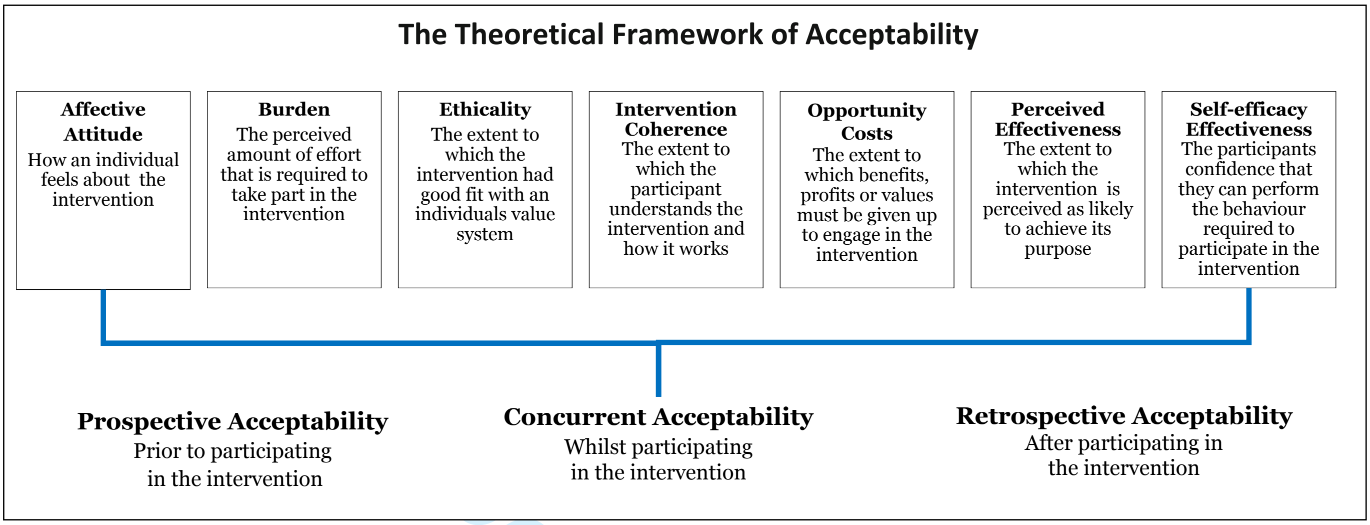

Figure 2, Participant characteristics by time point

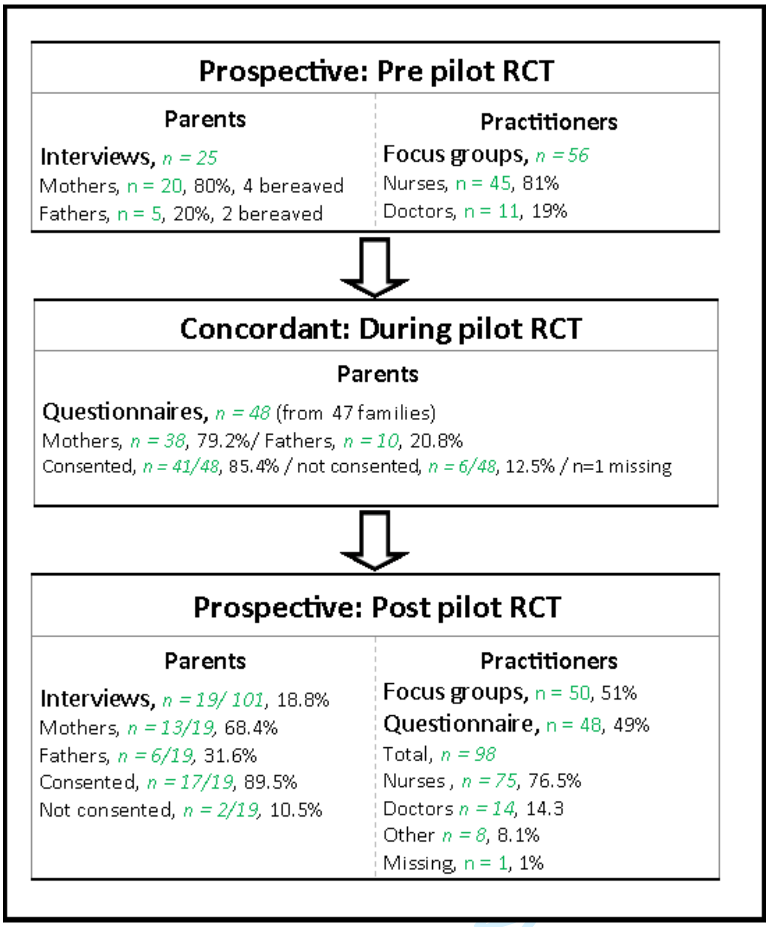

Figure 3, the Adapted Theoretical Framework of Acceptability

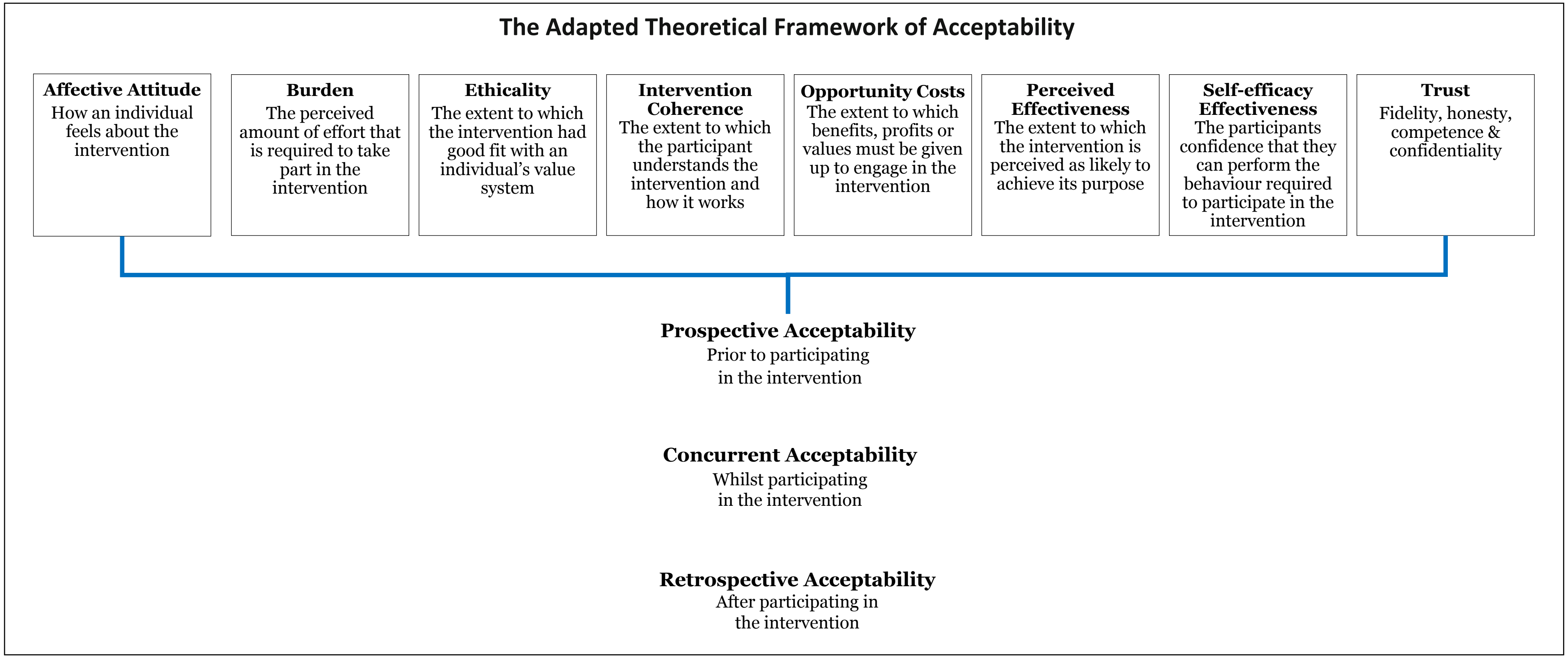

COREQ (COnsolidated criteria for REporting Qualitative research) Checklist

A checklist of items that should be included in reports of qualitative research. You must report the page number in your manuscript where you consider each of the items listed in this checklist. If you have not included this information, either revise your manuscript accordingly before submitting or note N/A.

Topic	Item No.	Guide Questions/Description	Reported on Page No.
**Domain 1: Research team and reflexivity**			
Personal characteristics			
Interviewer/facilitator	1	Which author/s conducted the interview or focus group?	
Credentials	2	What were the researcher's credentials? E.g. PhD, MD	
Occupation	3	What was their occupation at the time of the study?	
Gender	4	Was the researcher male or female?	
Experience and training	5	What experience or training did the researcher have?	
Relationship with participants			
Relationship established	6	Was a relationship established prior to study commencement?	
Participant knowledge of the interviewer	7	What did the participants know about the researcher? e.g. personal goals, reasons for doing the research	
Interviewer characteristics	8	What characteristics were reported about the inter viewer/facilitator? e.g. Bias, assumptions, reasons and interests in the research topic	
**Domain 2: Study design**			
Theoretical framework			
Methodological orientation and Theory	9	What methodological orientation was stated to underpin the study? e.g. grounded theory, discourse analysis, ethnography, phenomenology, content analysis	
Participant selection			
Sampling	10	How were participants selected? e.g. purposive, convenience, consecutive, snowball	
Method of approach	11	How were participants approached? e.g. face-to-face, telephone, mail, email	
Sample size	12	How many participants were in the study?	
Non-participation	13	How many people refused to participate or dropped out? Reasons?	
Setting			
Setting of data collection	14	Where was the data collected? e.g. home, clinic, workplace	
Presence of non-participants	15	Was anyone else present besides the participants and researchers?	
Description of sample	16	What are the important characteristics of the sample? e.g. demographic data, date	
Data collection			
Interview guide	17	Were questions, prompts, guides provided by the authors? Was it pilot tested?	
Repeat interviews	18	Were repeat inter views carried out? If yes, how many?	
Audio/visual recording	19	Did the research use audio or visual recording to collect the data?	
Field notes	20	Were field notes made during and/or after the inter view or focus group?	
Duration	21	What was the duration of the inter views or focus group?	
Data saturation	22	Was data saturation discussed?	
Transcripts returned	23	Were transcripts returned to participants for comment and/or	

For peer review only

Topic	Item No.	Guide Questions/Description	Reported on Page No.
		correction?	
**Domain 3: analysis and findings**			
Data analysis			
Number of data coders	24	How many data coders coded the data?	
Description of the coding tree	25	Did authors provide a description of the coding tree?	
Derivation of themes	26	Were themes identified in advance or derived from the data?	
Software	27	What software, if applicable, was used to manage the data?	
Participant checking	28	Did participants provide feedback on the findings?	
Reporting			
Quotations presented	29	Were participant quotations presented to illustrate the themes/findings? Was each quotation identified? e.g. participant number	
Data and findings consistent	30	Was there consistency between the data presented and the findings?	
Clarity of major themes	31	Were major themes clearly presented in the findings?	
Clarity of minor themes	32	Is there a description of diverse cases or discussion of minor themes?	

Developed from: Tong A, Sainsbury P, Craig J. Consolidated criteria for reporting qualitative research (COREQ): a 32-item checklist for interviews and focus groups. *International Journal for Quality in Health Care*. 2007. Volume 19, Number 6: pp. 349 – 357

Once you have completed this checklist, please save a copy and upload it as part of your submission. DO NOT include this checklist as part of the main manuscript document. It must be uploaded as a separate file.

For peer review only

BMJ Open

Establishing and augmenting views on the acceptability of a paediatric critical care randomised controlled trial (FEVER trial): a mixed methods study

Journal:	*BMJ Open*
Manuscript ID	bmjopen-2020-041952.R1
Article Type:	Original research
Date Submitted by the Author:	11-Jan-2021

Complete List of Authors:	Deja, Elizabeth; University of Liverpool, Department of Public Health, Policy and Systems<br>Peters, Mark; Great Ormond Street Hospital For Children NHS Trust, Paediatric Intensive Care Unit; University College London, Institute of Child Health<br>Khan, Imran ; Queen Mary University of London, Institute of Population Health Sciences<br>Mouncey, Paul; ICNARC, Clinical Trials Unit<br>Agbeko, Rachel; Great North Children's Hospital, Paediatric Intensive Care Unit<br>Fenn, Blaise; Patient partner<br>Watkins, Jason; Patient partner<br>Ramnarayan , Padmanabhan; Great Ormond Street Hospital for Children, Children's Acute Transport Service,<br>Tibby, Shane; Evelina Childrens Hospital, Paediatric Intensive Care<br>Thorburn, Kentigern; Alder Hey ChilDr.ens Hospital, Paediatric Intensive Care<br>Tume, Lyvonne; University of Salford, School of Health and Society<br>Rowan, Kathryn; Intensive Care National Audit and Research Centre (ICNARC),<br>Woolfall, Kerry; University of Liverpool,

**Primary Subject Heading**:	Intensive care
Secondary Subject Heading:	Qualitative research, Research methods, Paediatrics
Keywords:	Paediatric intensive & critical care < INTENSIVE & CRITICAL CARE, PAEDIATRICS, PAIN MANAGEMENT, QUALITATIVE RESEARCH

SCHOLARONE™
Manuscripts

I, the Submitting Author has the right to grant and does grant on behalf of all authors of the Work (as defined in the below author licence), an exclusive licence and/or a non-exclusive licence for contributions from authors who are: i) UK Crown employees; ii) where BMJ has agreed a CC-BY licence shall apply, and/or iii) in accordance with the terms applicable for US Federal Government officers or employees acting as part of their official duties; on a worldwide, perpetual, irrevocable, royalty-free basis to BMJ Publishing Group Ltd ("BMJ") its licensees and where the relevant Journal is co-owned by BMJ to the co-owners of the Journal, to publish the Work in this journal and any other BMJ products and to exploit all rights, as set out in our licence.

The Submitting Author accepts and understands that any supply made under these terms is made by BMJ to the Submitting Author unless you are acting as an employee on behalf of your employer or a postgraduate student of an affiliated institution which is paying any applicable article publishing charge ("APC") for Open Access articles. Where the Submitting Author wishes to make the Work available on an Open Access basis (and intends to pay the relevant APC), the terms of reuse of such Open Access shall be governed by a Creative Commons licence – details of these licences and which Creative Commons licence will apply to this Work are set out in our licence referred to above.

Other than as permitted in any relevant BMJ Author's Self Archiving Policies, I confirm this Work has not been accepted for publication elsewhere, is not being considered for publication elsewhere and does not duplicate material already published. I confirm all authors consent to publication of this Work and authorise the granting of this licence.

For peer review only

Establishing and augmenting views on the acceptability of a paediatric critical care randomised controlled trial (FEVER trial): a mixed methods study

Elizabeth Deja[1] PhD, Mark J Peters PhD [2], Imran Khan MSc[3] Paul R Mouncey MSc,[3] Rachel Agbeko FFICM,[4] Blaise Fenn,[5] Jason Watkins,[5] Padmanabhan Ramnarayan FFICM,[6] Shane M Tibby MB.ChB,[7] Kentigern Thorburn MMed MD,[8] Lyvonne N Tume PhD,[9] Kathryn M Rowan PhD [3] and, Kerry Woolfall PhD[1] on behalf of the Paediatric Intensive Care Society Study Group (PICS-SG)

Affiliations:
1 Institute of Population Health Sciences, University of Liverpool, Liverpool, United Kingdom
2 Respiratory, Critical Care and Anaesthesia Unit, University College London, Great Ormond Street, Institute of Child Health, London, United Kingdom
3 Institute of Population Health Sciences, Queen Mary University of London, United Kingdom
4 Paediatric Intensive Care Unit, Great North Children's Hospital, The Newcastle upon Tyne Hospitals NHS Foundation Trust, Institute of Cellular Medicine, Newcastle University, Newcastle, United Kingdom
5 Patient partner
6 Children's Acute Transport Service, Great Ormond Street Hospital, London, UK
7 Guy's & St Thomas' NHS Foundation Trust, Evelina Children's Hospital
8 Paediatric Intensive Care Unit, Alder Hey Children's Hospital, Liverpool, UK
9 Faculty of Health and Applied Sciences, University of the West of England, Glenside Campus, Bristol, United Kingdom

Address correspondence to:

Dr Elizabeth Deja. University of Liverpool, 1st Floor, Block B, Waterhouse Building, Liverpool, L69 3GL. Email: E.Deja@liverpool.ac.uk. Telephone: 0151 794 5613

Word count: 4008

For peer review only - http://bmjopen.bmj.com/site/about/guidelines.xhtml

ABSTRACT

Objective: To explore parent and staff views on the design of a randomised controlled trial investigating temperature thresholds for antipyretic intervention in critically ill children with fever and infection (the FEVER trial) during a multi-phase pilot study.

Design: Mixed methods study with data collected at three time points: 1) before, 2) during and 3) after a pilot trial.

Setting: English, Paediatric Intensive Care Unities (PICU).

Participants: 1) pre-pilot trial focus groups with pilot site staff (n=56) and interviews with parents(n=25) whose child had been admitted to PICU in the last three years with a Fever and suspected infection; 2) questionnaires with parents of randomised children following pilot trial recruitment (n=48 from 47 families) 3) post-pilot trial interviews with parents (n=19), focus groups (n=50) and a survey (n=48) with site staff. Analysis drew on Sekhon et al's (2017) theoretical framework of acceptability.

Results: There was initial support for the trial, yet some held concerns regarding the proposed temperature thresholds and not using paracetamol for pain or discomfort. Pre-trial findings informed protocol changes and training which influenced views on trial acceptability. Staff trained by the FEVER team found the trial more acceptable than those trained by colleagues. Parents and staff found the trial acceptable. Some concerns about pain or discomfort during weaning from ventilation remained.

Conclusions: Pre-trial findings and pilot trial experience influenced acceptability, providing insight into how challenges may be overcome. We present an adapted theoretical framework of acceptability to inform future trial feasibility studies.

KEY WORDS

Acceptability, Clinical trial, feasibility, Paediatric intensive care, qualitative, sepsis, practitioner training

ARTICLE SUMMARY

Strengths and limitations of this study

- The longitudinal design enabled collection of data from parents and staff with relevant experience before, during and after the pilot trial.
- The mixed methods approach including interviews, focus groups and surveys enabled breadth and depth of insight to help establish trial feasibility.
- Use of the Sekhon, Cartwright and Francis (2017) Theoretical Framework of Acceptability allowed trial acceptability to be evaluated as a multifaceted construct as opposed to a poorly defined binary (acceptable/not acceptable) approach.
- Data collected during the pilot trial stage were limited to parent perspectives, the majority of whom were mothers, although staff views were sought retrospectively.

For peer review only - http://bmjopen.bmj.com/site/about/guidelines.xhtml

INTRODUCTION

Recruitment and retention in clinical trials is a significant challenge, which leads to underpowered trials and the continued use of healthcare interventions that are not informed by robust scientific evidence [1-3]. Paediatric clinical trials are particularly challenging to conduct due to ethical and practical considerations that are not applicable to trials in adult settings [4-8]. For example, the eligible population is smaller and consent is obtained by proxy through children's parents or legal guardians [4, 9-11]. These considerations are compounded in critical care settings by the emotive and time sensitive situation in which they take place. Clinical trials must be acceptable to parents and health care practitioners to facilitate recruitment, adherence and consent [12, 13]. Sekhon, Cartwright and Francis (2017)[13] present a Theoretical Framework of Acceptability (TFA) (see Figure 1) to assist researchers in assessing the acceptability of healthcare interventions, including clinical trials. The TFA presents seven theoretical constructs for researchers to consider when assessing whether people delivering or receiving a healthcare intervention consider it to be appropriate. The constructs highlight considerations when establishing acceptability, such as how an individual feels about the healthcare intervention, the perceived burden of taking part and the extent to which the participant understands the healthcare intervention and how it works.

[INSERT FIGURE 1: Sekhon, Cartwright and Francis (2017) Theoretical Framework of Acceptability]

There is a lack of robust evidence or guidance to inform the management of fever due to an infection in critically ill children [14, 15]. The FEVER feasibility study aimed to establish whether it is possible to conduct a hospital based clinical trial comparing a comparing a permissive approach (treat as ≥40°C) with a restrictive approach (treat at $\geq$37.5^{o}C) to fever management in children. Perceived challenges to the successful conduct of a fever randomised control trial (RCT) included: a protocol that was likely to differ from usual clinical practice; potential parental and staff concerns about allowing a child's fever to rise without treatment; no time to seek informed consent [16, 17]; and the possibility that children may die before trial participation is discussed with parents.

The FEVER feasibility study involved pre-trial research with parents and staff, an observation of UK practice and a subsequent pilot RCT with embedded research exploring the perspectives of parents and staff involved in the pilot RCT. Pilot RCT and observation study findings are reported separately [18-20]. This paper focuses on research exploring parent and staff perspectives on trial acceptability drawing on the TFA.

For peer review only - http://bmjopen.bmj.com/site/about/guidelines.xhtml

METHODS

Study design

As part of the wider FEVER feasibility study (see Figure 2) we conducted mixed methods research involving interviews, focus groups and surveys with parents who had relevant experience and staff involved in the pilot RCT The research reported in this paper was conducted before, during and after the pilot RCT and aimed to explore parent and staff views on proposed FEVER trial including trial acceptability, design of information materials, temperature thresholds and the use of research without prior consent). The pilot RCT took place over a 3 month period (October to December 2017). Children were randomly allocated (1 : 1) using research without prior consent (RWPC) to permissive (39.5 °C) or restrictive (37.5 °C) temperature thresholds for antipyretics during their PICU stay while mechanically ventilated [19]. We used previous research that had explored patient and staff perspectives on trials conducted in paediatric emergency and critical care in the NHS [21-23] to develop topic guides for interviews and focus groups, questionnaires and participant information sheets (PIS).

[INSERT FIGURE 2: Fever Feasibility study design]

Patient and public involvement

Two parents (CF and JW) and one young adult (BF) with experience of severe infection and admission to hospital were co-investigators and members of the Study Management Group. They provided valuable input into the design and conduct of the study, including reviewing documents for parent interviews (e.g. draft pilot trial participant information sheets) and informing study recruitment approaches (i.e. identification of social media groups and charities). They were also involved in the review of study progress and findings**.**

Pre pilot trial: prospective recruitment and conduct

Parent interviews

English speaking parents of children (under 16 years) that had been admitted to an intensive care unit with a fever and infection in the last three years were recruited via: a database from a previous relevant study [24]; a letter from study sites [19]; and advertising on relevant social media and at sites. Some leniency was allowed if the child was admitted close to three years prior to interview (e.g. 3 years and two months). All routes invited parents to register interest in participation by contacting the research team.

For peer review only - http://bmjopen.bmj.com/site/about/guidelines.xhtml

Psychologist ED (PhD, female research associate) responded to parents' requests to participate in sequential order and checked eligibility. A draft pilot RCT PIS was emailed to parents prior to interview which included an outline of the study and current practice on the management of fever in critically ill children. ED conducted in person or via telephone based on parent preference. Audio-recorded verbal or written consent was sought before interviews as appropriate. Audio consent involved reading each aspect of the consent form to parents, including consent for audio recording and to receive a copy of the findings when the study is complete. Each box was initialled on the consent form when verbal consent was provided. Informed consent discussions were audio recorded for auditing purposes. Interviewing stopped when data saturation [25] and variation in sample was reached.

Staff focus groups

Co-investigators at the four pilot RCT sites [19] disseminated invitations to all staff who would be involved in the conduct of a clinical trial within a paediatric intensive care unit . KW (PhD, female, social scientist, Senior Lecturer) or ED provided a Participant information sheet and obtained written informed consent before the focus group began. The topic guide consisted of a mix of open and closed- ended questions. Closed - ended questions were administered using a voting system (Turning Technologies, Youngstown, OH, USA .This allowed for the collection of staff demographic information, to ensure data collection from all staff on key questions, such as views on trial acceptability. The use of Turning Point also enabled us to show grouped findings for closed questions on a screen to explore reasons for views in more depth verbally during the discussion.

Pilot RCT: Concurrent and retrospective recruitment and conduct

Interim analysis of prospective data informed subsequent topic guides and questionnaires.

Parent questionnaire and interviews

As part of the pilot RCT consent discussions, site researchers asked both parents if they would like to complete the FEVER consent questionnaire after the pilot RCT recruitment discussion (concurrent) and/or take part in a telephone interview approximately a month later (retrospective). In addition to collecting minimal demographic information the consent questionnaire asked them to indicate how strongly they agreed or disagreed with twelve statements about the Fever RCT followed by tick box and open- ended responses regarding their consent decision. ED contacted those who consented to interview in sequential order (by receipt of a consent form), stratifying by study arm (lower/higher temperature threshold) as the study progressed ensuring parents whose children had been randomised to both trial arms were represented in the sample.

For peer review only - http://bmjopen.bmj.com/site/about/guidelines.xhtml

Staff survey and focus groups

At the end of the pilot RCT, ED repeated focus groups with staff at the four pilot RCT sites to explore their experiences of pilot trial conduct and views on the proposed trial acceptability. Those unable to attend a focus group were invited by email to complete an online questionnaire containing the same closed- ended questions administered to focus group participants using the Turning Technologies (Youngstown, OH, USA) voting system.

Analysis

Digital audio recordings were transcribed verbatim by a professional transcription company (Voicescript Ltd., Bristol, UK). Transcripts were anonymised and checked for accuracy.

Table 1: Approach to thematic qualitative data analysis

Phase	Description
1. Familiarising with data	ED and KW read and re-read transcripts noting down initial ideas on themes.
2. Generating initial codes	Two complementary data-coding frameworks were developed (1 focus group data (KW), 1 interview data (ED) using *a priori* codes identified from the project proposal and topic guilds. During the familiarisation stage ED and KW identified additional data-driven codes and concepts not previously captured in the initial coding frame.
3. Developing the coding framework	KW coded 10% of the interview transcripts using the initial coding frame and made notes on any new themes identified and how the framework could be refined. In turn ED coded 10% of the focus group transcripts following the same procedure.
4. Defining and naming themes	Following review and reconciliation revised coding frames were subsequently developed and ordered into themes.
5. Completion of coding of transcripts	ED completed coding interview transcripts and KW completed coding focus group transcripts in preparation for write-up.
6. Producing the report	ED and KW developed the original manuscript using themes to relate back to the study aims ensuring key findings and recommendations were relevant to the FEVER trial design and site staff training (i.e. catalytic validity).Final discussion and development of selected themes occurred during the write-up phase.

For peer review only - http://bmjopen.bmj.com/site/about/guidelines.xhtml

ED and KW used a thematic analysis approach [26] to explore themes within the data related to views on trial design and acceptability (see table 1) . Analysis was interpretive and iterative [26, 27] . NVivo 10 software (QSR International Pty Ltd., Melbourne, Australia) was used to assist in the organisation and coding of qualitative data. Quantitative data from the parent and staff questionnaires were entered into SPSS Version 20.0 (IBM Corp., Armonk, NY, US) and analysed using descriptive statistics. Please see separate publication for further details [19]. ED and KW then synthesized data and used framework analysis [28] to map findings onto each component of the TFA by time point [13] (see tables 2-4). Where illustrative quotes are provided the participant identifier relates to each participant (e.g. P01 is participant 1).

For peer review only

For peer review only - http://bmjopen.bmj.com/site/about/guidelines.xhtml

Table 2: Prospective Acceptability of a FEVER Pilot Trial mapped to Sekhon et al's acceptability framework (2017)

Group & data collection method	Affective attitude	Burden	Ethicality	Intervention Coherence	Opportunity Costs	Perceived Effectiveness	Self-Efficacy
**Parents**<br><br>Interviews	100% stated they would consent for their child to take part in a Fever RCT<br><br>Would consent with a 40°C threshold, but 39.5-39.9°C more acceptable<br>*"I think 39.5 [°C]. But again you guys know best I'm just saying …that's very hot.* (P17, father, non-bereaved)	The intervention was not invasive	Belief it is important to help other children in the future.<br><br>Use of RWPC is necessary *"'I understand there's not really another way you can do it"* (P01, mother, non-bereaved)	Logically 'make sense' (P02, mother, bereaved).<br>PIS "it's simple for them to read. (P06, mother, bereaved)	Children would still be given all other kinds of care/ interventions	When study rationale was explained parents understood how allowing a fever could have a positive impact: *"Fever is meant to be like part of a fighting off, healing process isn't it?"* (P07, mother, non-bereaved)	The intervention was something parents understood and said they could support Important to approach for RWPC when parents have the capacity to make an informed decision
		Concerns about unnecessary discomfort /pain in higher threshold		Many suggested changes to the PIS to assist understanding and decision making	Concerns about loss of non-antipyretics effects of paracetamol e.g. reducing risk of seizures/riggers, pain relief.		
**Staff**<br><br>Focus group	82% (45/55, one missing) indicated 39.5°C was an acceptable permissive temperature threshold.<br>18.2% suggested 40°C was acceptable.<br>Only 20.4% suggested 37.5°C was acceptable as may lead to unnecessary intervention.	Watching a child be in pain or experience negative side effects: *"Incredibly difficult to wait and watch"* (P05, Staff, FG5).<br>The trial would be more acceptable if limited to ventilated children.	Mixed views on RWPC, n=25/49, (51%) thought acceptable based on past experience and the emergency situation.	Understanding that optimal temperature thresholds are unknown	Concerns about the loss of non-antipyretic effects e.g. discomfort relief, reducing risk of seizures/riggers, decreased cardio work load.	Evidence to support the trial*: "Well there is, there is a bit of science which suggests we should let the temperature get higher"* (P01, Staff, FG3)	No perceived issues with taking a temperature. Query method that is going to be used.
			Concerns about use of RWPC for an intervention that may not be supported by parents.	Want more clinical evidence as it goes against experiential knowledge (e.g. administering antipyretic at 38°C)	Staff with no experience of RWPC had concerns it would negatively impact on trust and the "*working relationship*" (P03, Staff, FG1)	Waiting for the permissive threshold would go against their clinical training or "*gut instinct*" (P05, Staff, FG2)	Nurses stated they may not follow the protocol if a child was upset, combative, in discomfort

Table 2 Key: shaded fields highlight potentially unacceptable aspects of the trial. *Abbreviations*: Research Without Prior Consent (RWPC), Focus Group (FG), Randomised Control Trial (RCT)

For peer review only - http://bmjopen.bmj.com/site/about/guidelines.xhtml

Table 3: Parent concordant acceptability of a FEVER Pilot Trial mapped to Sekhon et al's acceptability framework (2017)

Data collection method	Affective attitude	Burden	Ethicality	Intervention Coherence	Opportunity Costs	Perceived Effectiveness	Self-Efficacy
**Parents**<br><br>Questionnaire	N= 92/100 (92%) consented to taking part in the Fever Pilot RCT.	Not collected at this Time point	N= 32/41 (79%) reported the belief medical research studies are important<br><br>N= 42/48 (89.4%) satisfied with the RWPC process in the Fever pilot RCT<br><br>N= 32/41 (78%) selected helping other children as a reason for taking part.	N= 46/48, (96%) agreed that the information received about the Fever pilot RCT was clear & straightforward to understand	*My child is comfortable*" (P49, questionnaire, mother, permissive)<br><br>Concerns about their child being in pain or discomfort and impact on pre-existing medical condition.<br>*"My son had too many underlying medical conditions and felt it may hinder his recovery as he was selected to the upper limit before treatment* (P73, questionnaire, father, permissive)	N= 30/41 (73.2%) selected helping my child as a reason for taking part.<br><br>*"So far recovering well"* P21,mother,non-bereaved)	N= 41,(89.4%) felt they made the decision for their child to take part in the pilot trial

Table 3 Key: shaded fields highlight potentially unacceptable aspects of the trial. *Abbreviations*: Randomised Control Trial (RCT), Research Without Prior Consent (RWPC)

Table 4 Key: shaded fields highlight potentially unacceptable aspects of the trial. *Abbreviations:* Research Without Prior Consent (RWPC), Focus Group (FG), Randomised Control Trial (RCT)

For peer review only - http://bmjopen.bmj.com/site/about/guidelines.xhtml

Table 4: Retrospective Acceptability of a FEVER Pilot Trial mapped to Sekhon et al's acceptability framework (2017)

Group & data collection method(s)	Affective attitude	Burden	Ethicality	Intervention Coherence	Opportunity Costs	Perceived Effectiveness	Self-Efficacy
**Parents**<br>Interviews	*'I think it's a brilliant idea, so I'm all, I'm all for it"* (P80, interview mother, permissive).<br>Decliners also approve the trial *"They seem to be quick let's give them paracetamol. we've always been like a kind of, a hold off [. . .] I think sometimes paracetamol can hide other things going on as well."* (P83, interview, mother, permissive, decliner)	Not invasive *" I mean if it was more of an invasive study, I might have had to query it a little bit more but I was happy with everything"* (Parent 53, interview, father, restrictive).<br><br>Not in discomfort while ventilated<br><br>When being weaned of venerator some children displayed discomfort / distress.	Not causing harm to their child: *"I just thought, There's no harm in letting her into the study"* (P78, interview, mother, restrictive).<br><br>Wish to help others "if it's gonna help other people then yeah "(P85, interview, father, restrictive).<br><br>RWPC was acceptable	Involved treatment, which parents were familiar with; that logically made sense; that was *"Clearly explained"* (P74, interview, mother, permissive),	37.5°C ,very acceptable as normal practice at home<br><br>39.5°C acceptable if child is not in discomfort<br><br>≥39.5 °C not acceptable if the child is in pain or distress.	Understand how a fever may logically help a child.<br><br>Concerns about negative impact due to child's pre-existing medical conditions	Valued ability to withdraw or decline consent. *"I was happy enough for him to undergo the trial but if at any point the nurses thought he could do with the Calpol, or I thought he could, then I wanted the trial to stop it could do."* (P49, interview, mother, permissive)
**Staff**<br><br>Focus group & Survey	85 % (n=81/95) trial acceptable<br><br>95% (n=95/100, four missing) 37. °C acceptable<br><br>53% (n=42/79) 39.5°C acceptable	If the child is not conscious or not in pain no burden	Parents think the trial is acceptable therefore it is ethical to randomise their children.<br><br>89.4 % (n= 42/48) satisfied with the use of RWPC	If trained by trial team. Then 39.5°C acceptable	Mixed views, some observed no negative side effects and reported no costs.	Seeing as believing, *"It's a really good, valuable study to see on a larger scale"* (P04, Staff, FG5).	Valued being involved in trial design. Ability to follow the protocol mixed, n= 52/96 (54%) *"technically very easy to follow"*(*P06,* Staff, FG1)

For peer review only

For peer review only - http://bmjopen.bmj.com/site/about/guidelines.xhtml

RESULTS

Participant characteristics

Prospective (*Pre-pilot RCT*)*:* 25 semi-structured interviews (n=20 mothers, n=5 fathers) with bereaved (n=6) and non-bereaved (n=19) parents (see figure 3). Parents were interviewed a median of 14 months (range 6-38 months) after admission. Interviews took a median of 48 minutes (range 15-105 minutes). The 15-minute interview was concluded part way through by a bereaved father. Fifty-six staff took part in six focus groups across the four sites, lasting a median of 50 minutes (range 31-59 minutes). Staff mainly self-identified as nurses (n=45, 81%), all were involved in the clinical care of children.

[INSERT FIGURE 3: Participant characteristics by time point]

Concurrent (*During-pilot RCT*)*:* Eighty parents of the 100 children randomised to the pilot RCT consented to receive questionnaire, of these, 48 from 47 families completed and returned a questionnaire while their child was admitted to hospital. Of these, 41/48 (85%) provided consent and 6/48 (13%) declined consent (n=1 missing).

Retrospective (*Post-pilot RCT*)*:* Sixty- six parents of the 100 children randomised to the FEVER pilot trial consented to be contacted for an interview. Data saturation [25] was reached after eight interviews with parents of children allocated to the restrictive (lower) temperature threshold and after 11 interviews with parents of children allocated to the permissive (higher) temperature threshold. Parents were interviewed a median of 31 days after randomisation (range 9-70 days). Their children had received treatment for respiratory illness (e.g. bronchiolitis and respiratory syncytial virus) (n=18/19, 94%), cancer (n=1/19, 5%) and septic shock (n=1/19, 5%). Interviews took an average 32 minutes (range 20-50 minutes).

The staff sample included 98 site staff across all four pilot RCT sites. Almost half (48/98, 49 %) completed the questionnaire, with the rest attending a focus group. The majority (n=75, 77%) were nurses, n=45 (60%) were senior-level staff and most (n=79/98, 81%) were involved in the clinical care of children. Focus groups took an average of 53 minutes (range 23-106 minutes).

Pre-trial, prospective acceptability

All parents interviewed described how they would hypothetically consent for the use of their child's information in the proposed trial. Parents' views on trial acceptability appeared to be influenced by factors including: all other treatments for infection are given (*Opportunity costs*); the non-invasive nature of the intervention (*Burden*); support for RWPC in this context (*Ethicality*)*;* trust in medical

For peer review only - http://bmjopen.bmj.com/site/about/guidelines.xhtml

staff to act in the best interests of their child; and a belief that the trial question made sense and therefore likely to achieve its purpose (*Perceived effectiveness*):

> *"'cause fever is meant to be like part of a fighting off, healing process isn't it? A natural one… I can understand exactly why it would be interesting to see what happens."* (*P07, mother, non-bereaved*)

Although analysis of pre-RCT data indicated that many of the constructs of acceptability were met (see Table 2), there were also aspects of burden, opportunity costs, ethicality and intervention coherence identified as problematic by both parents and staff. As shown in Table 2, staff concerns outweighed support at this stage.

The majority of parents were not worried about the proposed restrictive temperature threshold of 37.5°C. However, staff expressed concerns that this was too low a threshold to administer an antipyretic (n=43/54, 80%, two missing) and would go against perceived "*normal practice*" (P01, Staff, FG4). A common concern was that children would be given unnecessary treatments in a clinical context where "*we try and give the minimum amount of drugs*" (P02, Staff, FG5) (*Affective attitude*).

In contrast, many parents voiced concerns about the acceptability of the permissive threshold with regards to increased risk of "*seizures*" (P03, mother, non-bereaved) and other potential detrimental side effects (opportunity costs), such as "*organs shutting down"* (P07, mother, non-bereaved), "*rigger*"" (P06, mother, bereaved) or unnecessary discomfort (*Burden*). The majority suggested that the pilot RCT would be more acceptable if the permissive temperature threshold was slightly lower (e.g. 39.9°C or 39.5°C). Although parents stated that they would still consent to a trial involving a threshold of 40°C (*Affective attitude*), as they trusted staff to monitor their child and act in their best interests:

> "*I would trust that my child was being monitored, it's not like they're waiting for her condition to get worse before they do something, you are having, a nurse by your bedside at all times, I had complete trust*." (P25, mother, non-bereaved)

Staff also described how a permissive threshold of ≥40°C was too high and how they would be concerned about not using paracetamol for analgesia in the less unwell, spontaneously breathing patients, who may be in pain or discomfort (*Burden*). In addition, staff were concerned about parental acceptability of the permissive threshold, RWPC (*Ethicality, Opportunity costs*) and the impact of increased cardiac workload (*Opportunity costs*).

Both groups understood the aims of the proposed trial. However, in addition to changes to temperature thresholds, amendments to the protocol were suggested. Parents identified aspects of

For peer review only - http://bmjopen.bmj.com/site/about/guidelines.xhtml

the PIS that required clarification, including whether not treating a temperature could cause a seizure, incorporating an explanation of how all other treatments would still be given. Staff requested additional information about the scientific evidence underpinning the research question, as well as clarification on key issues, such as what cooling methods could be used.

Response to pre-trial findings

Pre-trial findings were used in conjunction with observation study findings [19] to develop the pilot RCT protocol and site training. These included: a permissive temperature threshold of ≥39.5°C; inclusion criteria that required patients to be mechanically ventilated, therefore likely to be on other analgesia and changes to information materials. For example, staff training and PIS incorporated evidence to demonstrate how fever does not cause seizures and observation study findings that showed the restrictive temperature threshold (≥37.5°C) falls within usual practice. To address staff concerns about how parents may respond to trial and RWPC discussions, parent perspectives were communicated in site training, highlighting parental acceptability of RWPC, temperature thresholds, parents' questions about the study, and suggestions on how to address such questions.

Concurrent acceptability

As demonstrated in Table 3, parent questionnaire data showed that the six constructs of acceptability measured during this time point were met. Parents reported that the study information '*was clear and straightforward to understand*' (*Intervention coherence*). Ninety two percent of randomised children received consent for their continued participation in the pilot RCT (Affective attitude) [19]. Main reasons for providing consent related to the belief that participation might help their child (n=30/41, 73%, *Perceived effectiveness*) and help other children in the future (n=32/41, 78%, *Ethicality*). Parents also found the study acceptable because "*my child was comfortable*" (P49, questionnaire, mother, permissive) (*Opportunity costs and Burden*).

Of the eight that refused consent to continue, seven (88%) had been allocated to the permissive (higher) treatment group. Data suggested that parents who declined some element of their child participation still supported the proposed Fever RCT. Reasons for refusal of consent were linked to pre-existing medical conditions and the wish to limit any discomfort experienced by their child: *"My son had too many underlying medical conditions and felt it may hinder his recovery as he was selected to the upper limit"* (P73, questionnaire, father, permissive). Suggesting that there were still some concerns about withholding analgesia (*Opportunity costs*).

Staff acceptability was not measured at this point.

For peer review only - http://bmjopen.bmj.com/site/about/guidelines.xhtml

Post-trial, retrospective acceptability

Interviews conducted with parents a median one-month post randomisation supported and provided further insights into questionnaire findings. All seven constructs of acceptability were met (see table 4). Parents were interested in the trial question and felt the proposed trial was important (*Affective attitude, Perceived effectiveness*). Parents described staff as approaching them appropriately, with well-timed, clear, comprehensive study information leading to strong intervention coherence:

> *"I understood what they were saying and was happy to sort of go ahead, with the trial. If it wasn't explained to me too well, I probably wouldn't have bothered doing it."* (P77, interview, father, restrictive) (*Intervention coherence*)

Parents of children allocated to the restrictive temperature threshold found the trial very acceptable as giving paracetamol at this temperature was "*something that I would do myself anyway"* (P82, interview, father, restrictive) (*Ethicality*). Parents also viewed the permissive threshold to be acceptable. However, this acceptability was conditional on their child not being in discomfort (*Opportunity costs*):

> *"The only thing would be if she wasn't on any other kind of pain relief, but there's other things to manage, her discomfort".* (P73, interview, father, permissive)

Indeed, two mothers described how they found the trial acceptable and gave full consent, but later chose to withdraw their child from the study when they were being weaned from ventilation and sedation due to concerns about their child being in pain or distress. Parents valued the ability to withdraw or decline consent (*Self-efficacy*). They also described how they trusted staff to act in their child's best interests, including not adhering to the protocol by administering an antipyretic if at any point staff felt that it was needed (*Burden and Opportunity costs*):

> *"I know if anything did happen, you's can stop at any time. Stop it if they saw it was getting out of hand and he, and I felt like it, it wasn't helping, that I would stop it....they wouldn't let him go to the stage of him getting poorly"*. (P85, interview, father, restrictive)

Unlike parents' views, which largely remained consistent across study time points, staff perceptions of the acceptability of the lower temperature shifted during the course of the pilot RCT. Witnessing patient's positive reactions to RWPC and trial discussions and an awareness that ≥37.5°C was usual practice, resulted in 95% (n=95/100, four missing) of staff rating the restrictive threshold as acceptable or very acceptable: "*Everybody that was in the lower end of it, I found were like happy to take part*" (P01, Staff, FG4).

For peer review only - http://bmjopen.bmj.com/site/about/guidelines.xhtml

Staff had mixed views about the acceptability of the permissive temperature threshold. Approximately half (n=42/79, 53%) indicated that the ≥39.5°C threshold was acceptable. They valued how the trial team responded to their pre-trial concerns by changing the inclusion criteria to omit non-ventilated children (*Self-efficacy*). Some stated that their previous concerns about high temperatures causing harm or discomfort (*Opportunity costs*) and parents having a negative response to the trial and RWPC (*Ethicality*) were not observed:

> *"Some patients are randomised into the higher temperature and people see that they're actually manageable and it doesn't cause them any harm… It's kind of seeing is believing."* (P03, Staff, FG1)

Staff who did not find the permissive temperature acceptable were concerned about not giving paracetamol for pain or discomfort when a child was conscious (*Opportunity costs*). These concerns meant that some staff administered paracetamol before a child's temperature had reached ≥39.5 °C: *"I feel like potentially we're making our patients more uncomfortable."* (P01, Staff, FG2)

Interestingly, staff trained by their local unit colleagues were significantly more likely to find the permissive threshold not acceptable when compared to those trained directly by the pilot trial team ($X^2(2) = 8.78$, $p = 0.012$). Staff trained colleagues also rated site training as being poor (n=11/97, 11%). These staff remained unclear about the scientific rationale for the study and had lower Intervention coherence.

Despite issues with aspects of *Intervention coherence* and *Opportunity costs*, overall staff rated the fever trial acceptable (n=81/95, 85 %, *Affective attitude*) and practicable to conduct (n=80/95, 84%, *Self-efficacy*). Findings suggest that their views could be further augmented if the proposed Fever RCT protocol was revised to also exclude patients receiving non-invasive forms of ventilation (e.g. high-flow nasal oxygen) or those close to being extubated when sedation is being weaned.

Trust

During data analysis, we found that the concept of trust between parents and staff was prevalent within our data and intrinsically linked to trial acceptability. For example, parents found the trial acceptable because they trusted staff to put the needs of their child before the requirements of the study. Both groups discussed the trust parents place in medical expertise during a very emotive situation. Staff also highlighted that maintaining parental trust impacts on their decisions "*I feel like there's an element of trust there that would be broken from my point of view*." (P01, Staff, FG2,

For peer review only - http://bmjopen.bmj.com/site/about/guidelines.xhtml

retrospective). The construct of 'Trust' is not reflected within the TFA. We present an adapted TFA in Figure 4 incorporating Trust.

[INSERT FIGURE 4: The adapted Theoretical framework of Acceptability]

DISCUSSION

Our study highlights the value of conducting pre-trial research with key stakeholders to inform the design of challenging clinical trials [29]. Research with parents and staff, helped establish trial acceptability, as well as influence and changed perspectives over time. Prospective qualitative research identified mixed staff views, whilst parents found the trial broadly acceptable. Both the parental and staff support for RWPC in time critical trials is constant with previous research [24, 30-34]. Aspects of *Intervention Coherence, Opportunity Costs, Ethicality and Burden* [13] were identified that threatened trial success. The majority of staff concerns related to not using paracetamol or active cooling for pain relief, or to prevent febrile seizures [18]. Prospective findings informed changes to the PIS, staff training package and the addition of mechanical ventilation to inclusion criteria. Data from the concurrent and retrospective time points showed a positive response to such changes, particularly amongst staff. Suggestions to further augment views on trial acceptability and reduce the number of potential protocol deviations and withdrawals were identified. These include changes to trial inclusion criteria as well as staff training content and delivery [18].

Our findings demonstrate Sekhon and Francis' (2017) assertion that the acceptability of healthcare interventions is not a fixed construct. If we had taken a binary (acceptable/ not acceptable) or snapshot approach to determining acceptability, then we would not have been able to identify and address key concerns that threatened trial acceptability and ultimately, trial feasibility. The TFA was demonstrated to be comprehensive and relevant to our work. However, we found that the concept of trust between parents and staff was closely linked to trial acceptability and is not reflected in the framework. The importance of trust is a recurring theme in healthcare and medicine but is particularly salient in paediatric trials, as the more vulnerable the population, the greater the need for trust [5, 35]. Drawing on Hall et al's (2001) [35] work into defining trust in medical relationships, we propose the addition of an eighth construct of 'Trust' to help inform future trial feasibility research (see Figure 4). Further research is needed to test the adapted model in establishing the feasibility of other healthcare interventions and settings. This work will help to establish the appropriateness of trust as additional construct in the TFA.

As the pilot trial was conducted in three months, during the busy winter period the concurrent work only included parents and therefore lacks insight into staff perspectives during pilot trial conduct.

For peer review only - http://bmjopen.bmj.com/site/about/guidelines.xhtml

This limitation was compensated for by the use of retrospective (1 week-1 month) mixed methods ensuring a larger sample, through the survey and depth of insights, through focus groups. Insight was gained into the views of 8 (2 interviews 6 questionnaires) out of 18 parents (44%) who had declined their child's continued participation in one or more aspect of the pilot RCT. In particular, the interviews with parents who declined consent and nursing staff who found the protocol challenging to follow provided valuable information to assist with refining the study process for a definitive RCT. However, it is unknown whether or not the predominantly positive views of the declining parents who took part in an interview or questionnaire were shared by other parents who declined the FEVER pilot RCT.

In summary challenges to delivering the proposed trial included staff and parent concerns about the acceptability of the proposed protocol. Pre-trial research, staff training and experience of pilot trial conduct augmented views, providing insight into how challenges may be overcome, such as changes to the inclusion criteria and delivery of site training. We present an adapted TFA to inform the design of future trial feasibility studies.

Acknowledgements

The authors would like to thank all the parents who shared their experiences with us; their contribution to the research is invaluable. The authors are grateful to all the staff at participating UK hospitals and charities/ support groups for their help with recruitment and the UK PICS Study Group (PICS-SG) for their contributions to this study. We would also like to thank Ms Clara Francis (CF) as co-investigator and patient partner.

Funding

This study was funded by the UK National Institute for Health Research Health Technology Assessment programme (15/44/01) and supported by the National Institute for Health Research Great Ormond Street Hospital Biomedical Research Centre. The trial sponsor was Great Ormond Street Hospital NHS Foundation Trust, Joint R&D Office GOSH/ICH, 30 Guilford Street, London WC1N 1EH, United Kingdom (email: Research.Governance@gosh.nhs.uk). The Clinical Trials Unit for the Fever study was the ICNARC CTU. The views expressed in this publication are those of the authors and not necessarily of the funder, the sponsor, the NHS, the NIHR, or the UK Department of Health.

Ethics approval and consent to participate

Ethical approval for the study was provided by the National Research Ethics Committee (NRES) (17/LO/1139). Management approvals were obtained from all study sites.

For peer review only - http://bmjopen.bmj.com/site/about/guidelines.xhtml

Data statement

The datasets generated during and/or analysed during the current study are not publicly available as consent was not sought for data sharing.

Competing interests' statement.

MJP is a member of the NIHR HTA Board. KMR is a member of the NIHR HS & DR Board.

Author contributions

Dr Elizabeth Deja conducted the qualitative research, co-analysed the data, drafted the initial manuscript, reviewed and revised the manuscript.

Professor Mark J Peters is the chief investigator of the FEVER study, he conceived, designed and oversaw conduct of the FEVER study and critically reviewed the manuscript.

Mr Imran Khan was the study manager at the ICNARC CTU responsible for day to day FEVER study management and critically reviewed the manuscript.

Mr Paul R Mouncey is the head of research at ICNARC CTU, a co-applicant, involved in the design and coordination of the FEVER study, contributed to and reviewed the manuscript.

Mr Blaise Fenn and Mr Jason Watkins were study co-applicants, patient and parent representatives who contributed to the design and conduct of the FEVER study including mixed methods perspectives elements.

Dr Padmanabhan Ramnarayan co-applicant helped design and conduct the FEVER study.

Dr Rachel Agbeko, Dr Shane M Tibby, Professor Lyvonne N Tume were co-applicants, site principal investigators and critically reviewed the manuscript.

Dr Kentigern Thorburn site principal investigator and critically reviewed the manuscript.

Dr Kathryn M Rowan is the director at ICNARC CTU, study co-applicant involved in designing and overseeing the FEVER study.

Dr Kerry Woolfall was a co-applicant on the FEVER study, designed the mixed methods perspectives elements of the study, supervised the qualitative research, co- analysed the data and reviewed and revised the manuscript.

Licence statement

I, the Submitting Author has the right to grant and does grant on behalf of all authors of the Work (as defined in the below author licence), an exclusive licence and/or a non-exclusive licence for contributions from authors who are: i) UK Crown employees; ii) where BMJ has agreed a CC-BY licence shall apply, and/or iii) in accordance with the terms applicable for US Federal Government officers or employees acting as part of their official duties; on a worldwide, perpetual, irrevocable, royalty-free basis to BMJ Publishing Group Ltd ("BMJ") its licensees and where the relevant Journal is co-owned by BMJ to the co-owners of the Journal, to publish the Work in BMJ Open and any other BMJ products and to exploit all rights, as set out in our licence.

REFRENCES

For peer review only - http://bmjopen.bmj.com/site/about/guidelines.xhtml

1. McDonald AM, Knight RC, Campbell MK, et al. What influences recruitment to randomised controlled trials? A review of trials funded by two UK funding agencies. Trials. 2006;7(9).
2. Bower P, Brueton V, Gamble C, et al. Interventions to improve recruitment and retention in clinical trials: a survey and workshop to assess current practice and future priorities. Trials. 2014;15(399).
3. Treweek S, Mitchell E, Pitkethly M, et al. Strategies to improve recruitment to randomised controlled trials. Cochrane Database Syst Re. 2010.
4. Caldwell P, Murphy S, Butow P, Craig J. Clinical trials in children The Lancet. 2004;364(9436):803-11.
5. Greenberg RG, Gamel B, Bloom D, et al. Parents' perceived obstacles to pediatric clinical trial participation: Findings from the clinical trials transformation initiative Contemporary Clinical Trials Communications. 2018.
6. Sammons HM, Starkey ES. Ethical issues of clinical trials in children. Paediatrics and child health 2011;22(2).
7. Field MJ, Behrman RE. Institute of Medicine (U.S.). Committee on Clinical Research Involving Children., Ethical Conduct of Clinical Research Involving Children. Washington, D.C.: National Academies Press; 2004.
8. Naka F, Strober B, Shahriari M. Clinical trials: Kids are not just little people. Clinics in Dermatology. 2017;35(6):583-93.
9. Caldwell PHY, Butow PN, Craig JC. Pediatricians' attitudes towards randomized controlled trials involving children. The Journal of Pediatrics 2002;141(6).
10. Caldwell P, Butow P, Craig J. Parents' attitudes to randomised controlled trials involving children. Journal of Pediatricts 2003;145:555–60.
11. Enpr-EMA. Preparedness of medicines' clinical trials in paediatrics. Amsterdam: European Medicines Agency; 2020.
12. Diepeveen S, Ling T, Suhrcke M, Roland M, Marteau TM. Public acceptability of government intervention to change health-related behaviours: a systematic review and narrative synthesis. BMC Public health 2013;13(756).
13. Sekhon M, Cartwright M, Francis JJ. Acceptability of healthcare interventions: an overview of reviews and development of a theoretical framework. BMC Health Services Research. 2017;17(88).
14. Brick T, Agbeko RS, Davies P, et al. Too hot to handle: a survey of attitudes towards fever of 462 pediatric intensive care unit staff. Eur J Pediatr 2016;176:423–7.
15. NICE. Fever in Under 5s: Assessment and Initial Management. In: guideline N, editor. London: National Institute for Health and Care Excellence; 2019. p. 1-36.
16. Proposal for a Regulation of the European Parliament and of the Council on Clinical trials on medicinal products for human use, and repealing Directive 2001/20/EC., (2013).
17. The Medicines for human use (Clinical trials) and Blood Safety and Quality (Amendment) Regulations., (2008).
18. Peters MJ, Woolfall K, Khan I, et al. Permissive versus restrictive temperature thresholds in critically ill children with fever and infection: a multicentre randomized clinical pilot trial. Critical Care. 2019;23(1):69.
19. Peters MJ, Woolfall K, Khan I, et al. Different temperature thresholds for antipyretic intervention in critically ill children with fever due to infection: the FEVER feasibility RCT. Health Technology Assessment 2019;23(5):1 - 148.
20. Woolfall K, O'Hara C, Deja E, et al. Parents' prioritised outcomes for trials investigating treatments for paediatric severe infection: a qualitative synthesis. Arch Dis Child. 2019;0:1-6.
21. Woolfall K, Frith L, Gamble C, et al. How parents and practitioners experience research without prior consent (deferred consent) for emergency research involving children with life threatening conditions: a mixed method study. BMJ Open. 2015;5.
22. Inwald DP, Canter R, Woolfall K, et al. Restricted fluid bolus volume in early septic shock: results of the Fluids in Shock pilot trial. Arch Dis Child. 2018.

For peer review only

For peer review only - http://bmjopen.bmj.com/site/about/guidelines.xhtml

23. Molyneux S, Njue M, Boga M, et al. The words will pass with the blowing wind': staff and parent views of the deferred consent process, with prior assent, used in an emergency fluids trial in two African hospitals. PLOS ONE 2013;8(8).
24. Inwald D, Canter R, Woolfall K, et al. Restricted fluid bolus versus current practice in children with septic shock: the FiSh feasibility study and pilot RCT. Health Technol Assess. 2018;22(51).
25. Saunder B, Sim J, Kingstone T, et al. Saturation in qualitative research: exploring its conceptualization and operationalization. Qual Quant. 2018;52(4).
26. Braun V, Clarke V. Using thematic analysis in psychology. Qualitative research in psychology. 2006;3(2):77-101.
27. Braun V, Clarke V. What can "thematic analysis''offer health and wellbeing researchers? International journal of qualitative studies on health and well-being. 2014;9:26152.
28. Gale NK, Heath G, Cameron E, Rashid S, Redwood S. Using the framework method for the analysis of qualitative data in multi-disciplinary health research. BMC Medical Research Methodology. 2013;13(117).
29. O'Cathain A, Thomas KJ, Drabble SJ, Rudolph A, Goode J, Hewison J. Maximising the value of combining qualitative research and randomised controlled trials in health research: the QUAlitative Research in Trials (QUART) study--a mixed methods study. Health Technol Assess. 2014;18(38):1-197, v-vi.
30. Canvin K, Jacoby A. Duty, desire or indifference? A qualitative study of patient decisions about recruitment to an epilepsy treatment trial. Trials. 2006;7(32).
31. Roper L, Sherratt FC, Young B, et al. Children's views on research without prior consent in emergency situations: a UK qualitative study. BMJ open 2018;8.
32. Waterfield T, Lyttle MD, Shields M, et al. Parents' and clinicians' views on conducting paediatric diagnostic test accuracy studies without prior informed consent: qualitative insight from the Petechiae in Children study (PiC). Archives of Disease in Childhood. 2019:317.
33. Woolfall K, Frith L, Dawson A, Gamble C, Lyttle MD, Young B. Fifteen-minute consultation: an evidence-based approach to research without prior consent (deferred consent) in neonatal and paediatric critical care trials. Archives of disease in childhood - Education and amp; practice edition. 2016;101(1):49-53.
34. Brierley J, Larcher V. Emergency research in children: options for ethical recruitment. Journal of Medical Ethics. 2011;37(7):429-32.
35. Hall MA, Dugan E, Zheng B, Mishra AK. Trust in Physicians and Medical Institutions:What Is It, Can It Be Measured,and Does It Matter? The Milbank Quarterly. 2001;79(4).

For peer review only

For peer review only - http://bmjopen.bmj.com/site/about/guidelines.xhtml

LEDGENDS

Figure 1: Sekhon, Cartwright and Francis (2017) Theoretical Framework of Acceptability

Figure 2: Fever Feasibility study design

Figure 3: Participant characteristics by time point

Table 1: Approach to qualitative data analysis

Table 2: Prospective Acceptability of a FEVER Pilot Trial mapped to Sekhon et al's acceptability framework (2017)

Table 3: Parent concordant acceptability of a FEVER Pilot Trial mapped to Sekhon et al's acceptability framework (2017)

Table 4: Respective Acceptability of a FEVER Pilot Trial mapped to Sekhon et als's acceptability framework (2017)

Figure 4: The adapted Theoretical framework of Acceptability

For peer review only

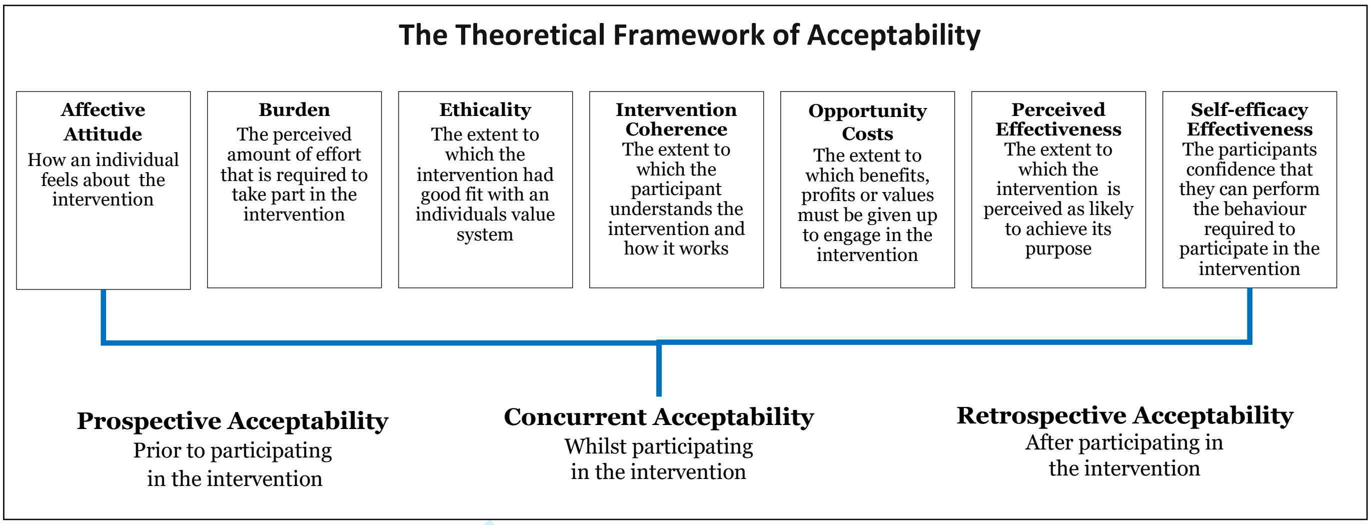

peer review only

Stage 1

Pre trial feasibility research including:

- Interviews with Parents with experience their child being admitted to an intensive care unit with a fever and suspected infection in the preceding 3 years
- Focus groups with clinicians (nurses and doctors) working the 4 PICUs planned to be included in the pilot
- Observational study of UK practice related to fever management*

Stage 2

- Pilot RCT in 4 hospitals comparing a permissive approach (treat as $\geq 40^{\circ}$C) with a restrictive approach (treat at $\geq 37.5^{\circ}$C) to fever management in children*
- Embedded survey to explore parent perspectives at the point of trial recruitment.

Stage 3

- Interviews with parents of children randomised to the pilot RCT approximately one month after hospital discharge.
- Survey and focus groups with staff involved in the pilot RCT at the end of trial recruitment.

*reported separately

For peer review only - http://bmjopen.bmj.com/site/about/guidelines.xhtml

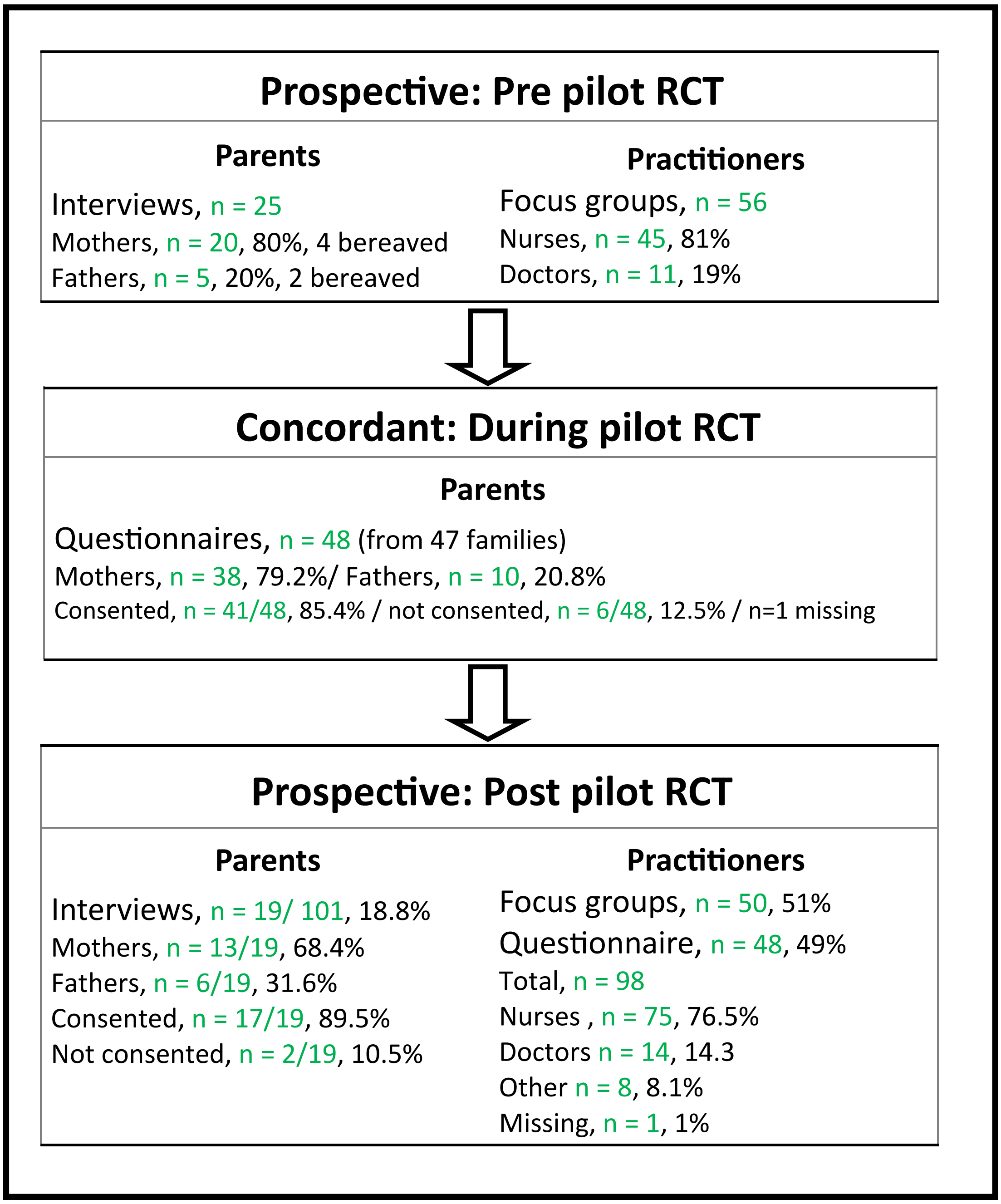
Prospective: Pre pilot RCT
Parents
Interviews, n = 25
Mothers, n = 20, 80%, 4 bereaved
Fathers, n = 5, 20%, 2 bereaved
Practitioners
Focus groups, n = 56
Nurses, n = 45, 81%
Doctors, n = 11, 19%
Concordant: During pilot RCT
Parents
Questionnaires, n = 48 (from 47 families)
Mothers, n = 38, 79.2%/ Fathers, n = 10, 20.8%
Consented, n = 41/48, 85.4% / not consented, n = 6/48, 12.5% / n=1 missing
Prospective: Post pilot RCT
Parents
Interviews, n = 19/ 101, 18.8%
Mothers, n = 13/19, 68.4%
Fathers, n = 6/19, 31.6%
Consented, n = 17/19, 89.5%
Not consented, n = 2/19, 10.5%
Practitioners
Focus groups, n = 50, 51%
Questionnaire, n = 48, 49%
Total, n = 98
Nurses , n = 75, 76.5%
Doctors n = 14, 14.3
Other n = 8, 8.1%
Missing, n = 1, 1%

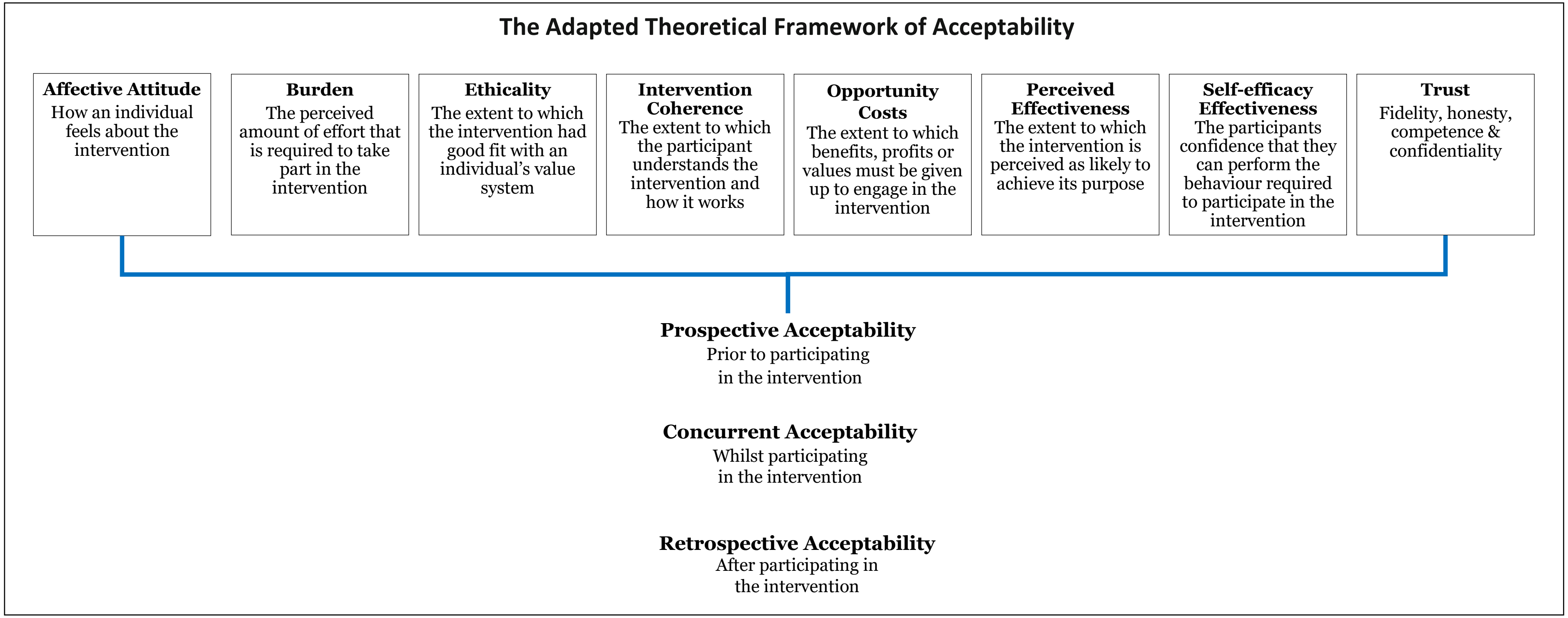
The Adapted Theoretical Framework of Acceptability
Affective Attitude
How an individual feels about the intervention
Burden
The perceived amount of effort that is required to take part in the intervention
Ethicality
The extent to which the intervention had good fit with an individual's value system
Intervention Coherence
The extent to which the participant understands the intervention and how it works
Opportunity Costs
The extent to which benefits, profits or values must be given up to engage in the intervention
Perceived Effectiveness
The extent to which the intervention is perceived as likely to achieve its purpose
Self-efficacy Effectiveness
The participants confidence that they can perform the behaviour required to participate in the intervention
Trust
Fidelity, honesty, competence & confidentiality
Prospective Acceptability
Prior to participating in the intervention
Concurrent Acceptability
Whilst participating in the intervention
Retrospective Acceptability
After participating in the intervention

COREQ (COnsolidated criteria for REporting Qualitative research) Checklist

A checklist of items that should be included in reports of qualitative research. You must report the page number in your manuscript where you consider each of the items listed in this checklist. If you have not included this information, either revise your manuscript accordingly before submitting or note N/A.

Topic	Item No.	Guide Questions/Description	Reported on Page No.
**Domain 1: Research team and reflexivity**			
Personal characteristics			
Interviewer/facilitator	1	Which author/s conducted the interview or focus group?	
Credentials	2	What were the researcher's credentials? E.g. PhD, MD	
Occupation	3	What was their occupation at the time of the study?	
Gender	4	Was the researcher male or female?	
Experience and training	5	What experience or training did the researcher have?	
Relationship with participants			
Relationship established	6	Was a relationship established prior to study commencement?	
Participant knowledge of the interviewer	7	What did the participants know about the researcher? e.g. personal goals, reasons for doing the research	
Interviewer characteristics	8	What characteristics were reported about the inter viewer/facilitator? e.g. Bias, assumptions, reasons and interests in the research topic	
**Domain 2: Study design**			
Theoretical framework			
Methodological orientation and Theory	9	What methodological orientation was stated to underpin the study? e.g. grounded theory, discourse analysis, ethnography, phenomenology, content analysis	
Participant selection			
Sampling	10	How were participants selected? e.g. purposive, convenience, consecutive, snowball	
Method of approach	11	How were participants approached? e.g. face-to-face, telephone, mail, email	
Sample size	12	How many participants were in the study?	
Non-participation	13	How many people refused to participate or dropped out? Reasons?	
Setting			
Setting of data collection	14	Where was the data collected? e.g. home, clinic, workplace	
Presence of non-participants	15	Was anyone else present besides the participants and researchers?	
Description of sample	16	What are the important characteristics of the sample? e.g. demographic data, date	
Data collection			
Interview guide	17	Were questions, prompts, guides provided by the authors? Was it pilot tested?	
Repeat interviews	18	Were repeat inter views carried out? If yes, how many?	
Audio/visual recording	19	Did the research use audio or visual recording to collect the data?	
Field notes	20	Were field notes made during and/or after the inter view or focus group?	
Duration	21	What was the duration of the inter views or focus group?	
Data saturation	22	Was data saturation discussed?	
Transcripts returned	23	Were transcripts returned to participants for comment and/or	

For peer review only

Topic	Item No.	Guide Questions/Description	Reported on Page No.
		correction?	
**Domain 3: analysis and findings**			
Data analysis			
Number of data coders	24	How many data coders coded the data?	
Description of the coding tree	25	Did authors provide a description of the coding tree?	
Derivation of themes	26	Were themes identified in advance or derived from the data?	
Software	27	What software, if applicable, was used to manage the data?	
Participant checking	28	Did participants provide feedback on the findings?	
Reporting			
Quotations presented	29	Were participant quotations presented to illustrate the themes/findings? Was each quotation identified? e.g. participant number	
Data and findings consistent	30	Was there consistency between the data presented and the findings?	
Clarity of major themes	31	Were major themes clearly presented in the findings?	
Clarity of minor themes	32	Is there a description of diverse cases or discussion of minor themes?	

Developed from: Tong A, Sainsbury P, Craig J. Consolidated criteria for reporting qualitative research (COREQ): a 32-item checklist for interviews and focus groups. *International Journal for Quality in Health Care*. 2007. Volume 19, Number 6: pp. 349 – 357

Once you have completed this checklist, please save a copy and upload it as part of your submission. DO NOT include this checklist as part of the main manuscript document. It must be uploaded as a separate file.

For peer review only

BMJ Open

Establishing and augmenting views on the acceptability of a paediatric critical care randomised controlled trial (FEVER trial): a mixed methods study

Journal:	*BMJ Open*
Manuscript ID	bmjopen-2020-041952.R2
Article Type:	Original research
Date Submitted by the Author:	02-Feb-2021
Complete List of Authors:	Deja, Elizabeth; University of Liverpool, Department of Public Health, Policy and Systems<br>Peters, Mark; Great Ormond Street Hospital For Children NHS Trust, Paediatric Intensive Care Unit; University College London, Institute of Child Health<br>Khan, Imran ; Queen Mary University of London, Institute of Population Health Sciences<br>Mouncey, Paul; ICNARC, Clinical Trials Unit<br>Agbeko, Rachel; Great North Children's Hospital, Paediatric Intensive Care Unit<br>Fenn, Blaise; Patient partner<br>Watkins, Jason; Patient partner<br>Ramnarayan , Padmanabhan; Great Ormond Street Hospital for Children, Children's Acute Transport Service,<br>Tibby, Shane; Evelina Childrens Hospital, Paediatric Intensive Care<br>Thorburn, Kentigern; Alder Hey ChilDr.ens Hospital, Paediatric Intensive Care<br>Tume, Lyvonne; University of Salford, School of Health and Society<br>Rowan, Kathryn; Intensive Care National Audit and Research Centre (ICNARC),<br>Woolfall, Kerry; University of Liverpool,
**Primary Subject Heading**:	Intensive care
Secondary Subject Heading:	Qualitative research, Research methods, Paediatrics, Ethics
Keywords:	Paediatric intensive & critical care < INTENSIVE & CRITICAL CARE, PAEDIATRICS, PAIN MANAGEMENT, QUALITATIVE RESEARCH, MEDICAL EDUCATION & TRAINING

SCHOLARONE™ Manuscripts

I, the Submitting Author has the right to grant and does grant on behalf of all authors of the Work (as defined in the below author licence), an exclusive licence and/or a non-exclusive licence for contributions from authors who are: i) UK Crown employees; ii) where BMJ has agreed a CC-BY licence shall apply, and/or iii) in accordance with the terms applicable for US Federal Government officers or employees acting as part of their official duties; on a worldwide, perpetual, irrevocable, royalty-free basis to BMJ Publishing Group Ltd ("BMJ") its licensees and where the relevant Journal is co-owned by BMJ to the co-owners of the Journal, to publish the Work in this journal and any other BMJ products and to exploit all rights, as set out in our licence.

The Submitting Author accepts and understands that any supply made under these terms is made by BMJ to the Submitting Author unless you are acting as an employee on behalf of your employer or a postgraduate student of an affiliated institution which is paying any applicable article publishing charge ("APC") for Open Access articles. Where the Submitting Author wishes to make the Work available on an Open Access basis (and intends to pay the relevant APC), the terms of reuse of such Open Access shall be governed by a Creative Commons licence – details of these licences and which Creative Commons licence will apply to this Work are set out in our licence referred to above.

Other than as permitted in any relevant BMJ Author's Self Archiving Policies, I confirm this Work has not been accepted for publication elsewhere, is not being considered for publication elsewhere and does not duplicate material already published. I confirm all authors consent to publication of this Work and authorise the granting of this licence.

For peer review only

Establishing and augmenting views on the acceptability of a paediatric critical care randomised controlled trial (FEVER trial): a mixed methods study

Elizabeth Deja[1] PhD, Mark J Peters PhD [2], Imran Khan MSc[3] Paul R Mouncey MSc,[3] Rachel Agbeko FFICM,[4] Blaise Fenn,[5] Jason Watkins,[5] Padmanabhan Ramnarayan FFICM,[6] Shane M Tibby MB.ChB,[7] Kentigern Thorburn MMed MD,[8] Lyvonne N Tume PhD,[9] Kathryn M Rowan PhD [3] and, Kerry Woolfall PhD[1] on behalf of the Paediatric Intensive Care Society Study Group (PICS-SG)

Affiliations:

1 Institute of Population Health Sciences, University of Liverpool, Liverpool, United Kingdom

2 Respiratory, Critical Care and Anaesthesia Unit, University College London, Great Ormond Street, Institute of Child Health, London, United Kingdom

3 Institute of Population Health Sciences, Queen Mary University of London, United Kingdom

4 Paediatric Intensive Care Unit, Great North Children's Hospital, The Newcastle upon Tyne Hospitals NHS Foundation Trust, Institute of Cellular Medicine, Newcastle University, Newcastle, United Kingdom

5 Patient partner

6 Children's Acute Transport Service, Great Ormond Street Hospital, London, UK

7 Guy's & St Thomas' NHS Foundation Trust, Evelina Children's Hospital

8 Paediatric Intensive Care Unit, Alder Hey Children's Hospital, Liverpool, UK

9 Faculty of Health and Applied Sciences, University of the West of England, Glenside Campus, Bristol, United Kingdom

Address correspondence to:

Dr Elizabeth Deja. University of Liverpool, 1st Floor, Block B, Waterhouse Building, Liverpool, L69 3GL. Email: E.Deja@liverpool.ac.uk. Telephone: 0151 794 5613

Word count: 4018

For peer review only

For peer review only - http://bmjopen.bmj.com/site/about/guidelines.xhtml

ABSTRACT

Objective: To explore parent and staff views on the acceptability of a randomised controlled trial investigating temperature thresholds for antipyretic intervention in critically ill children with fever and infection (the FEVER trial) during a multi-phase pilot study.

Design: Mixed methods study with data collected at three time points: 1) before, 2) during and 3) after a pilot trial.

Setting: English, Paediatric Intensive Care Units (PICU).

Participants: 1) pre-pilot trial focus groups with pilot site staff (n=56) and interviews with parents(n=25) whose child had been admitted to PICU in the last three years with a Fever and suspected infection; 2) questionnaires with parents of randomised children following pilot trial recruitment (n=48 from 47 families) 3) post-pilot trial interviews with parents (n=19), focus groups (n=50) and a survey (n=48) with site staff. Analysis drew on Sekhon et al's (2017) theoretical framework of acceptability.

Results: There was initial support for the trial, yet some held concerns regarding the proposed temperature thresholds and not using paracetamol for pain or discomfort. Pre-trial findings informed protocol changes and training which influenced views on trial acceptability. Staff trained by the FEVER team found the trial more acceptable than those trained by colleagues. Parents and staff found the trial acceptable. Some concerns about pain or discomfort during weaning from ventilation remained.

Conclusions: Pre-trial findings and pilot trial experience influenced acceptability, providing insight into how challenges may be overcome. We present an adapted theoretical framework of acceptability to inform future trial feasibility studies.

KEY WORDS

Acceptability, Clinical trial, feasibility, Paediatric intensive care, qualitative, sepsis, practitioner training

ARTICLE SUMMARY

Strengths and limitations of this study

- The longitudinal design enabled collection of data from parents and staff with relevant experience before, during and after the pilot trial.
- The mixed methods approach including interviews, focus groups and surveys enabled breadth and depth of insight to help establish trial feasibility.
- Use of the Sekhon, Cartwright and Francis (2017) Theoretical Framework of Acceptability allowed trial acceptability to be evaluated as a multifaceted construct as opposed to a poorly defined binary (acceptable/not acceptable) approach.
- Data collected during the pilot trial stage were limited to parent perspectives, the majority of whom were mothers, although staff views were sought retrospectively.

For peer review only - http://bmjopen.bmj.com/site/about/guidelines.xhtml

For peer review only

INTRODUCTION

Recruitment and retention in clinical trials is a significant challenge, which leads to underpowered trials and the continued use of healthcare interventions that are not informed by robust scientific evidence [1-3]. Paediatric clinical trials are particularly challenging to conduct due to ethical and practical considerations that are not applicable to trials in adult settings [4-8]. For example, the eligible population is smaller and consent is obtained by proxy through children's parents or legal guardians [4, 9-11]. These considerations are compounded in critical care settings by the emotive and time sensitive situation in which they take place. Clinical trials must be acceptable to parents and health care practitioners to facilitate recruitment, adherence and consent [12, 13]. Sekhon, Cartwright and Francis (2017)[13] present a Theoretical Framework of Acceptability (TFA) (see Figure 1) to assist researchers in assessing the acceptability of healthcare interventions, including clinical trials. The TFA presents seven theoretical constructs for researchers to consider when assessing whether people delivering or receiving a healthcare intervention consider it to be appropriate. The constructs highlight considerations when establishing acceptability, such as how an individual feels about the healthcare intervention, the perceived burden of taking part and the extent to which the participant understands the healthcare intervention and how it works.

[INSERT FIGURE 1: Sekhon, Cartwright and Francis (2017) Theoretical Framework of Acceptability]

There is a lack of robust evidence or guidance to inform the management of fever due to an infection in critically ill children [14, 15]. The FEVER feasibility study aimed to establish whether it is possible to conduct a hospital based clinical trial comparing a permissive approach (treat as ≥40°C) with a restrictive approach (treat at ≥37.5^{o}C) to fever management in children. Perceived challenges to the successful conduct of a fever randomised control trial (RCT) included: a protocol that was likely to differ from usual clinical practice; potential parental and staff concerns about allowing a child's fever to rise without treatment; no time to seek informed consent [16, 17]; and the possibility that children may die before trial participation is discussed with parents.

The FEVER feasibility study involved a multi-phase pilot study including: pre-trial research with parents and staff, an observation of UK practice and a subsequent pilot RCT with embedded research exploring the perspectives of parents and staff involved in the pilot RCT. The FEVER Pilot RCT (ISRCTN16022198) and FEVER observational study (NCT03028818) findings are reported separately [18-20]. This paper focuses on research exploring parent and staff perspectives on trial acceptability drawing on the TFA.

For peer review only - http://bmjopen.bmj.com/site/about/guidelines.xhtml

METHODS

Study design

As part of the wider FEVER feasibility study (see Figure 2) we conducted mixed methods research involving interviews, focus groups and surveys with parents who had relevant experience and staff involved in the pilot RCT. The research reported in this paper was conducted before, during and after the pilot RCT and aimed to explore parent and staff views on the proposed FEVER trial including trial acceptability, design of information materials, temperature thresholds and the use of research without prior consent (RWPC). The pilot RCT took place over a 3 month period (October to December 2017). Children were randomly allocated (1 : 1) without prior consent to permissive (39.5 °C) or restrictive (37.5 °C) temperature thresholds for antipyretics during their PICU stay while mechanically ventilated [19]. We used previous research that had explored patient and staff perspectives on trials conducted in paediatric emergency and critical care in the NHS [21-23] to develop topic guides for interviews and focus groups, questionnaires and participant information sheets (PIS).

[INSERT FIGURE 2: Fever Feasibility study design]

Patient and public involvement

Two parents (CF and JW) and one young adult (BF) with experience of severe infection and admission to hospital were co-investigators and members of the Study Management Group. They provided valuable input into the design and conduct of the study, including reviewing documents for parent interviews (e.g. draft pilot trial PIS) and informing study recruitment approaches (i.e. identification of social media groups and charities). They were also involved in the review of study progress and findings.

Pre pilot trial: prospective recruitment and conduct

Parent interviews

English speaking parents of children (under 16 years) that had been admitted to an intensive care unit with a fever and infection in the last three years were recruited via: a database from a previous relevant study [24]; a letter from study sites [19]; and advertising on relevant social media and at sites. Some leniency was allowed if the child was admitted close to three years prior to interview (e.g. 3 years and two months). All routes invited parents to register interest in participation by contacting the research team.

For peer review only - http://bmjopen.bmj.com/site/about/guidelines.xhtml

Psychologist ED (PhD, female research associate) responded to parents' requests to participate in sequential order and checked eligibility. A draft pilot RCT PIS was emailed to parents prior to interview which included an outline of the study and current practice on the management of fever in critically ill children. ED conducted interviews in person or via telephone based on parent preference. Audio-recorded verbal or written consent was sought before interviews as appropriate. Audio consent involved reading each aspect of the consent form to parents, including consent for audio recording and to receive a copy of the findings when the study is complete. Each box was initialled on the consent form when verbal consent was provided. Informed consent discussions were audio recorded for auditing purposes. Interviewing stopped when data saturation [25] and variation in sample was reached.

Staff focus groups

Co-investigators at the four pilot RCT sites [19] disseminated invitations to all staff who would be involved in the conduct of a clinical trial within a paediatric intensive care unit. KW (PhD, female, social scientist, Senior Lecturer) or ED provided a PIS and obtained written informed consent before the focus group began. The topic guide consisted of a mix of open and closed- ended questions. Closed - ended questions were administered using the Turning Technologies (Youngstown, OH, USA) voting system. .This allowed for the collection of staff demographic information, to ensure data collection from all staff on key questions, such as views on trial acceptability. The use of Turning Point also enabled us to show grouped findings for closed questions on a screen to explore reasons for views in more depth verbally during the discussion.

Pilot RCT: Concurrent and retrospective recruitment and conduct

Interim analysis of prospective data informed subsequent topic guides and questionnaires.

Parent questionnaire and interviews

As part of the pilot RCT consent discussions, site researchers asked both parents if they would like to complete the FEVER consent questionnaire after the pilot RCT recruitment discussion (concurrent) and/or take part in a telephone interview approximately a month later (retrospective). In addition to collecting minimal demographic information the consent questionnaire asked them to indicate how strongly they agreed or disagreed with twelve statements about the Fever RCT followed by tick box and open- ended responses regarding their consent decision. ED contacted those who consented to interview in sequential order (by receipt of a consent form), stratifying by study arm (lower/higher

For peer review only - http://bmjopen.bmj.com/site/about/guidelines.xhtml

temperature threshold) as the study progressed ensuring parents whose children had been randomised to both trial arms were represented in the sample.

Staff survey and focus groups

At the end of the pilot RCT, ED repeated focus groups with staff at the four pilot RCT sites to explore their experiences of pilot trial conduct and views on the proposed trial acceptability. Those unable to attend a focus group were invited by email to complete an online questionnaire containing the same closed- ended questions administered to focus group participants using the Turning Technologies (Youngstown, OH, USA) voting system.

Analysis

Digital audio recordings were transcribed verbatim by a professional transcription company (Voicescript Ltd., Bristol, UK). Transcripts were anonymised and checked for accuracy.

Table 1: Approach to thematic qualitative data analysis

Phase	Description
1. Familiarising with data	ED and KW read and re-read transcripts noting down initial ideas on themes.
2. Generating initial codes	Two complementary data-coding frameworks were developed (1 focus group data (KW), 1 interview data (ED) using *a priori* codes identified from the project proposal and topic guilds. During the familiarisation stage ED and KW identified additional data-driven codes and concepts not previously captured in the initial coding frame.
3. Developing the coding framework	KW coded 10% of the interview transcripts using the initial coding frame and made notes on any new themes identified and how the framework could be refined. In turn ED coded 10% of the focus group transcripts following the same procedure.
4. Defining and naming themes	Following review and reconciliation revised coding frames were subsequently developed and ordered into themes.
5. Completion of coding of transcripts	ED completed coding interview transcripts and KW completed coding focus group transcripts in preparation for write-up.
6. Producing the report	ED and KW developed the original manuscript using themes to relate back to the study aims ensuring key findings and recommendations were relevant to the FEVER trial design and site staff training (i.e. catalytic validity).Final discussion and

For peer review only - http://bmjopen.bmj.com/site/about/guidelines.xhtml

	development of selected themes occurred during the write-up phase.

ED and KW used a thematic analysis approach [26] to explore themes within the data related to views on trial design and acceptability (see table 1) . Analysis was interpretive and iterative [26, 27] . NVivo 10 software (QSR International Pty Ltd., Melbourne, Australia) was used to assist in the organisation and coding of qualitative data. Quantitative data from the parent and staff questionnaires were entered into SPSS Version 20.0 (IBM Corp., Armonk, NY, US) and analysed using descriptive statistics. Please see separate publication for further details [19]. ED and KW then synthesized data and used framework analysis [28] to map findings onto each component of the TFA by time point [13] (see tables 2-4). Where illustrative quotes are provided the participant identifier relates to each participant (e.g. P01 is participant 1).

For peer review only

For peer review only - http://bmjopen.bmj.com/site/about/guidelines.xhtml

Table 2: Prospective Acceptability of a FEVER Pilot Trial mapped to Sekhon et al's acceptability framework (2017)

Group & data collection method	Affective attitude	Burden	Ethicality	Intervention Coherence	Opportunity Costs	Perceived Effectiveness	Self-Efficacy
**Parents**<br><br>Interviews	100% stated they would consent for their child to take part in a Fever RCT<br><br>Would consent with a 40°C threshold, but 39.5-39.9°C more acceptable<br>*"I think 39.5 [°C]. But again you guys know best I'm just saying …that's very hot.* (P17, father, non-bereaved)	The intervention was not invasive<br><br>Concerns about unnecessary discomfort /pain in higher threshold	Belief it is important to help other children in the future.<br><br>Use of RWPC is necessary *"'I understand there's not really another way you can do it"* (P01, mother, non-bereaved)	Logically 'make sense' (P02, mother, bereaved).<br>PIS "it's simple for them to read. (P06, mother, bereaved)<br>Many suggested changes to the PIS to assist understanding and decision making	Children would still be given all other kinds of care/ interventions<br><br>Concerns about loss of non-antipyretics effects of paracetamol e.g. reducing risk of seizures/rigors, pain relief.	When study rationale was explained parents understood how allowing a fever could have a positive impact: *"Fever is meant to be like part of a fighting off, healing process isn't it?"* (P07, mother, non-bereaved)	The intervention was something parents understood and said they could support Important to approach for RWPC when parents have the capacity to make an informed decision
**Staff**<br><br>Focus group	82% (45/55, one missing) indicated 39.5°C was an acceptable permissive temperature threshold.<br>18.2% suggested 40°C was acceptable.<br>Only 20.4% suggested 37.5°C was acceptable as may lead to unnecessary intervention.	Watching a child be in pain or experience negative side effects: *"Incredibly difficult to wait and watch"* (P05, Staff, FG5).<br>The trial would be more acceptable if limited to ventilated children.	Mixed views on RWPC, n=25/49, (51%) thought acceptable based on past experience and the emergency situation.<br><br>Concerns about use of RWPC for an intervention that may not be supported by parents.	Understanding that optimal temperature thresholds are unknown<br><br>Want more clinical evidence as it goes against experiential knowledge (e.g. administering antipyretic at 38°C)	Concerns about the loss of non-antipyretic effects e.g. discomfort relief, reducing risk of seizures/rigors, decreased cardio work load.<br><br>Staff with no experience of RWPC had concerns it would negatively impact on trust and the "*working relationship*" (P03, Staff, FG1)	Evidence to support the trial*: "Well there is, there is a bit of science which suggests we should let the temperature get higher"* (P01, Staff, FG3)<br><br>Waiting for the permissive threshold would go against their clinical training or "*gut instinct*" (P05, Staff, FG2)	No perceived issues with taking a temperature. Query method that is going to be used.<br><br>Nurses stated they may not follow the protocol if a child was upset, combative, in discomfort

Table 2 Key: shaded fields highlight potentially unacceptable aspects of the trial. *Abbreviations*: Research Without Prior Consent (RWPC), Focus Group (FG), Randomised Control Trial (RCT), participant information sheets (PIS)

For peer review only - http://bmjopen.bmj.com/site/about/guidelines.xhtml

Table 3: Parent concordant acceptability of a FEVER Pilot Trial mapped to Sekhon et al's acceptability framework (2017)

Data collection method	Affective attitude	Burden	Ethicality	Intervention Coherence	Opportunity Costs	Perceived Effectiveness	Self-Efficacy
**Parents**<br><br>Questionnaire	N= 92/100 (92%) consented to taking part in the Fever Pilot RCT.	Not collected at this Time point	N= 32/41 (79%) reported the belief medical research studies are important<br><br>N= 42/48 (89.4%) satisfied with the RWPC process in the Fever pilot RCT<br><br>N= 32/41 (78%) selected helping other children as a reason for taking part.	N= 46/48, (96%) agreed that the information received about the Fever pilot RCT was clear & straightforward to understand	*My child is comfortable*" (P49, questionnaire, mother, permissive)<br><br>Concerns about their child being in pain or discomfort and impact on pre-existing medical condition.<br>*"My son had too many underlying medical conditions and felt it may hinder his recovery as he was selected to the upper limit before treatment* (P73, questionnaire, father, permissive)	N= 30/41 (73.2%) selected helping my child as a reason for taking part.<br><br>"*So far recovering well*" P21,mother,non-bereaved)	N= 41,(89.4%) felt they made the decision for their child to take part in the pilot trial

Table 3 Key: shaded fields highlight potentially unacceptable aspects of the trial. *Abbreviations*: Randomised Control Trial (RCT), Research Without Prior Consent (RWPC)

Table 4 Key: shaded fields highlight potentially unacceptable aspects of the trial. *Abbreviations:* Research Without Prior Consent (RWPC), Focus Group (FG), Randomised Control Trial (RCT)

For peer review only - http://bmjopen.bmj.com/site/about/guidelines.xhtml

Table 4: Retrospective Acceptability of a FEVER Pilot Trial mapped to Sekhon et al's acceptability framework (2017)

Group & data collection method(s)	Affective attitude	Burden	Ethicality	Intervention Coherence	Opportunity Costs	Perceived Effectiveness	Self-Efficacy
**Parents** Interviews	*'I think it's a brilliant idea, so I'm all, I'm all for it"* (P80, interview mother, permissive). Decliners also approve the trial *"They seem to be quick let's give them paracetamol. we've always been like a kind of, a hold off [. . .] I think sometimes paracetamol can hide other things going on as well."* (P83, interview, mother, permissive, decliner)	Not invasive *" I mean if it was more of an invasive study, I might have had to query it a little bit more but I was happy with everything"* (Parent 53, interview, father, restrictive).<br><br>Not in discomfort while ventilated<br><br>When being weaned of venerator some children displayed discomfort / distress.	Not causing harm to their child: *"I just thought, There's no harm in letting her into the study"* (P78, interview, mother, restrictive).<br><br>Wish to help others "if it's gonna help other people then yeah "(P85, interview, father, restrictive).<br><br>RWPC was acceptable	Involved treatment, which parents were familiar with; that logically made sense; that was *"Clearly explained"* (P74, interview, mother, permissive),	37.5°C ,very acceptable as normal practice at home<br><br>39.5°C acceptable if child is not in discomfort<br><br>≥39.5 °C not acceptable if the child is in pain or distress.	Understand how a fever may logically help a child.<br><br>Concerns about negative impact due to child's pre-existing medical conditions	Valued ability to withdraw or decline consent. *"I was happy enough for him to undergo the trial but if at any point the nurses thought he could do with the Calpol, or I thought he could, then I wanted the trial to stop it could do."* (P49, interview, mother, permissive)
**Staff**<br><br>Focus group & Survey	85 % (n=81/95) trial acceptable<br><br>95% (n=95/100, four missing) 37. °C acceptable<br><br>53% (n=42/79) 39.5°C acceptable	If the child is not conscious or not in pain no burden	Parents think the trial is acceptable therefore it is ethical to randomise their children.<br><br>89.4 % (n= 42/48) satisfied with the use of RWPC	If trained by trial team. Then 39.5°C acceptable	Mixed views, some observed no negative side effects and reported no costs.	Seeing as believing, *"It's a really good, valuable study to see on a larger scale"* (P04, Staff, FG5).	Valued being involved in trial design. Ability to follow the protocol mixed, n= 52/96 (54%) *"technically very easy to follow"*(*P06,* Staff, FG1)

For peer review only - http://bmjopen.bmj.com/site/about/guidelines.xhtml

RESULTS

Participant characteristics

Prospective (*Pre-pilot RCT*)*:* 25 semi-structured interviews (n=20 mothers, n=5 fathers) with bereaved (n=6) and non-bereaved (n=19) parents (see figure 3). Parents were interviewed a median of 14 months (range 6-38 months) after admission. Interviews took a median of 48 minutes (range 15-105 minutes). The 15-minute interview was concluded part way through by a bereaved father. Fifty-six staff took part in six focus groups across the four sites, lasting a median of 50 minutes (range 31-59 minutes). Staff mainly self-identified as nurses (n=45, 81%), all were involved in the clinical care of children.

[INSERT FIGURE 3: Participant characteristics by time point]

Concurrent (*During-pilot RCT*)*:* Eighty parents of the 100 children randomised to the pilot RCT consented to receive questionnaire, of these, 48 from 47 families completed and returned a questionnaire while their child was admitted to hospital. Of these, 41/48 (85%) provided consent and 6/48 (13%) declined consent (n=1 missing).

Retrospective (*Post-pilot RCT*)*:* Sixty- six parents of the 100 children randomised to the FEVER pilot trial consented to be contacted for an interview. Data saturation [25] was reached after eight interviews with parents of children allocated to the restrictive (lower) temperature threshold and after 11 interviews with parents of children allocated to the permissive (higher) temperature threshold. Parents were interviewed a median of 31 days after randomisation (range 9-70 days). Their children had received treatment for respiratory illness (e.g. bronchiolitis and respiratory syncytial virus) (n=18/19, 94%), cancer (n=1/19, 5%) and septic shock (n=1/19, 5%). Interviews took an average 32 minutes (range 20-50 minutes).

The staff sample included 98 site staff across all four pilot RCT sites. Almost half (48/98, 49 %) completed the questionnaire, with the rest attending a focus group. The majority (n=75, 77%) were nurses, n=45 (60%), were senior-level staff and most (n=79/98, 81%) were involved in the clinical care of children. Focus groups took an average of 53 minutes (range 23-106 minutes).

Pre-trial, prospective acceptability

All parents interviewed described how they would hypothetically consent for the use of their child's information in the proposed trial. Parents' views on trial acceptability appeared to be influenced by factors including: all other treatments for infection are given (*Opportunity costs*); the non-invasive nature of the intervention (*Burden*); support for RWPC in this context (*Ethicality*)*;* trust in medical

For peer review only - http://bmjopen.bmj.com/site/about/guidelines.xhtml

staff to act in the best interests of their child; and a belief that the trial question made sense and therefore likely to achieve its purpose (*Perceived effectiveness*):

> *"cause fever is meant to be like part of a fighting off, healing process isn't it? A natural one… I can understand exactly why it would be interesting to see what happens."* (*P07, mother, non-bereaved*)

Although analysis of pre-RCT data indicated that many of the constructs of acceptability were met (see Table 2), there were also aspects of burden, opportunity costs, ethicality and intervention coherence identified as problematic by both parents and staff. As shown in Table 2, staff concerns outweighed support at this stage.

The majority of parents were not worried about the proposed restrictive temperature threshold of 37.5°C. However, staff expressed concerns that this was too low a threshold to administer an antipyretic (n=43/54, 80%, two missing) and would go against perceived "*normal practice*" (P01, Staff, FG4). A common concern was that children would be given unnecessary treatments in a clinical context where "*we try and give the minimum amount of drugs*" (P02, Staff, FG5) (*Affective attitude*).

In contrast, many parents voiced concerns about the acceptability of the permissive threshold with regards to increased risk of "*seizures*" (P03, mother, non-bereaved) and other potential detrimental side effects (opportunity costs), such as "*organs shutting down"* (P07, mother, non-bereaved), "*rigor"*" (P06, mother, bereaved) or unnecessary discomfort (*Burden*). The majority suggested that the pilot RCT would be more acceptable if the permissive temperature threshold was slightly lower (e.g. 39.9°C or 39.5°C). Although parents stated that they would still consent to a trial involving a threshold of 40°C (*Affective attitude*), as they trusted staff to monitor their child and act in their best interests:

> "*I would trust that my child was being monitored, it's not like they're waiting for her condition to get worse before they do something, you are having, a nurse by your bedside at all times, I had complete trust.*" (P25, mother, non-bereaved)

Staff also described how a permissive threshold of $\geq$40°C was too high and how they would be concerned about not using paracetamol for analgesia in the less unwell, spontaneously breathing patients, who may be in pain or discomfort (*Burden*). In addition, staff were concerned about parental acceptability of the permissive threshold, RWPC (*Ethicality, Opportunity costs*) and the impact of increased cardiac workload (*Opportunity costs*).

Both groups understood the aims of the proposed trial. However, in addition to changes to temperature thresholds, amendments to the protocol were suggested. Parents identified aspects of

For peer review only - http://bmjopen.bmj.com/site/about/guidelines.xhtml

the PIS that required clarification, including whether not treating a temperature could cause a seizure, incorporating an explanation of how all other treatments would still be given. Staff requested additional information about the scientific evidence underpinning the research question, as well as clarification on key issues, such as what cooling methods could be used.

Response to pre-trial findings

Pre-trial findings were used in conjunction with observation study findings [19] to develop the pilot RCT protocol and site training. These included: a permissive temperature threshold of ≥39.5°C; inclusion criteria that required patients to be mechanically ventilated, therefore likely to be on other analgesia and changes to information materials. For example, staff training and PIS incorporated evidence to demonstrate how fever does not cause seizures and observation study findings that showed the restrictive temperature threshold (≥37.5°C) falls within usual practice. To address staff concerns about how parents may respond to trial and RWPC discussions, parent perspectives were communicated in site training, highlighting parental acceptability of RWPC, temperature thresholds, parents' questions about the study, and suggestions on how to address such questions.

Concurrent acceptability

As demonstrated in Table 3, parent questionnaire data showed that the six constructs of acceptability measured during this time point were met. Parents reported that the study information '*was clear and straightforward to understand*' (*Intervention coherence*). Ninety two percent of randomised children received consent for their continued participation in the pilot RCT (Affective attitude) [19]. Main reasons for providing consent related to the belief that participation might help their child (n=30/41, 73%, *Perceived effectiveness*) and help other children in the future (n=32/41, 78%, *Ethicality*). Parents also found the study acceptable because "*my child was comfortable*" (P49, questionnaire, mother, permissive) (*Opportunity costs and Burden*).

Of the eight that refused consent to continue, seven (88%) had been allocated to the permissive (higher) treatment group. Data suggested that parents who declined some element of their child participation still supported the proposed Fever RCT. Reasons for refusal of consent were linked to pre-existing medical conditions and the wish to limit any discomfort experienced by their child: *"My son had too many underlying medical conditions and felt it may hinder his recovery as he was selected to the upper limit"* (P73, questionnaire, father, permissive). Suggesting that there were still some concerns about withholding analgesia (*Opportunity costs*).

Staff acceptability was not measured at this point.

For peer review only - http://bmjopen.bmj.com/site/about/guidelines.xhtml

Post-trial, retrospective acceptability

Interviews conducted with parents a median one-month post randomisation supported and provided further insights into questionnaire findings. All seven constructs of acceptability were met (see table 4). Parents were interested in the trial question and felt the proposed trial was important (*Affective attitude, Perceived effectiveness*). Parents described staff as approaching them appropriately, with well-timed, clear, comprehensive study information leading to strong intervention coherence:

> *"I understood what they were saying and was happy to sort of go ahead, with the trial. If it wasn't explained to me too well, I probably wouldn't have bothered doing it."* (P77, interview, father, restrictive) (*Intervention coherence*)

Parents of children allocated to the restrictive temperature threshold found the trial very acceptable as giving paracetamol at this temperature was "*something that I would do myself anyway"* (P82, interview, father, restrictive) (*Ethicality*). Parents also viewed the permissive threshold to be acceptable. However, this acceptability was conditional on their child not being in discomfort (*Opportunity costs*):

> *"The only thing would be if she wasn't on any other kind of pain relief, but there's other things to manage, her discomfort".* (P73, interview, father, permissive)

Indeed, two mothers described how they found the trial acceptable and gave full consent, but later chose to withdraw their child from the study when they were being weaned from ventilation and sedation due to concerns about their child being in pain or distress. Parents valued the ability to withdraw or decline consent (*Self-efficacy*). They also described how they trusted staff to act in their child's best interests, including not adhering to the protocol by administering an antipyretic if at any point staff felt that it was needed (*Burden and Opportunity costs*):

> *"I know if anything did happen, you's can stop at any time. Stop it if they saw it was getting out of hand and he, and I felt like it, it wasn't helping, that I would stop it….they wouldn't let him go to the stage of him getting poorly"*. (P85, interview, father, restrictive)

Unlike parents' views, which largely remained consistent across study time points, staff perceptions of the acceptability of the lower temperature shifted during the course of the pilot RCT. Witnessing patient's positive reactions to RWPC and trial discussions and an awareness that ≥37.5°C was usual practice, resulted in 95% (n=95/100, four missing) of staff rating the restrictive threshold as acceptable or very acceptable: "*Everybody that was in the lower end of it, I found were like happy to take part*" (P01, Staff, FG4).

For peer review only - http://bmjopen.bmj.com/site/about/guidelines.xhtml

Staff had mixed views about the acceptability of the permissive temperature threshold. Approximately half (n=42/79, 53%) indicated that the ≥39.5°C threshold was acceptable. They valued how the trial team responded to their pre-trial concerns by changing the inclusion criteria to omit non-ventilated children (*Self-efficacy*). Some stated that their previous concerns about high temperatures causing harm or discomfort (*Opportunity costs*) and parents having a negative response to the trial and RWPC (*Ethicality*) were not observed:

> *"Some patients are randomised into the higher temperature and people see that they're actually manageable and it doesn't cause them any harm… It's kind of seeing is believing."* (P03, Staff, FG1)

Staff who did not find the permissive temperature acceptable were concerned about not giving paracetamol for pain or discomfort when a child was conscious (*Opportunity costs*). These concerns meant that some staff administered paracetamol before a child's temperature had reached ≥39.5 °C: *"I feel like potentially we're making our patients more uncomfortable."* (P01, Staff, FG2)

Interestingly, staff trained by their local unit colleagues were significantly more likely to find the permissive threshold not acceptable when compared to those trained directly by the pilot trial team ($X^2(2)$ =8.78, p = 0.012). Staff trained colleagues also rated site training as being poor (n=11/97, 11%). These staff remained unclear about the scientific rationale for the study and had lower Intervention coherence.

Despite issues with aspects of *Intervention coherence* and *Opportunity costs*, overall staff rated the fever trial acceptable (n=81/95, 85 %, *Affective attitude*) and practicable to conduct (n=80/95, 84%, *Self-efficacy*). Findings suggest that their views could be further augmented if the proposed Fever RCT protocol was revised to also exclude patients receiving non-invasive forms of ventilation (e.g. high-flow nasal oxygen) or those close to being extubated when sedation is being weaned.

Trust

During data analysis, we found that the concept of trust between parents and staff was prevalent within our data and intrinsically linked to trial acceptability. For example, parents found the trial acceptable because they trusted staff to put the needs of their child before the requirements of the study. Both groups discussed the trust parents place in medical expertise during a very emotive situation. Staff also highlighted that maintaining parental trust impacts on their decisions "*I feel like there's an element of trust there that would be broken from my point of view*." (P01, Staff, FG2,

For peer review only - http://bmjopen.bmj.com/site/about/guidelines.xhtml

retrospective). The construct of 'Trust' is not reflected within the TFA. We present an adapted TFA in Figure 4 incorporating Trust.

[INSERT FIGURE 4: The adapted Theoretical framework of Acceptability]

DISCUSSION

Our study highlights the value of conducting pre-trial research with key stakeholders to inform the design of challenging clinical trials [29]. Research with parents and staff helped establish trial acceptability, as well as influence and changed perspectives over time. Prospective qualitative research identified mixed staff views, whilst parents found the trial broadly acceptable. Both the parental and staff support for RWPC in time critical trials is constant with previous research [24, 30-34]. Aspects of *Intervention Coherence, Opportunity Costs, Ethicality and Burden* [13] were identified that threatened trial success. The majority of staff concerns related to not using paracetamol or active cooling for pain relief, or to prevent febrile seizures [18]. Prospective findings informed changes to the PIS, staff training package and the addition of mechanical ventilation to inclusion criteria. Data from the concurrent and retrospective time points showed a positive response to such changes, particularly amongst staff. Suggestions to further augment views on trial acceptability and reduce the number of potential protocol deviations and withdrawals were identified. These include changes to trial inclusion criteria as well as staff training content and delivery [18].

Our findings demonstrate Sekhon and Francis' (2017) assertion that the acceptability of healthcare interventions is not a fixed construct. If we had taken a binary (acceptable/ not acceptable) or snapshot approach to determining acceptability, then we would not have been able to identify and address key concerns that threatened trial acceptability and ultimately, trial feasibility. The TFA was demonstrated to be comprehensive and relevant to our work. However, we found that the concept of trust between parents and staff was closely linked to trial acceptability and is not reflected in the framework. The importance of trust is a recurring theme in healthcare and medicine but is particularly salient in paediatric trials, as the more vulnerable the population, the greater the need for trust [5, 35]. Drawing on Hall et al's (2001) [35] work into defining trust in medical relationships, we propose the addition of an eighth construct of 'Trust' to help inform future trial feasibility research (see Figure 4). Further research is needed to test the adapted model in establishing the feasibility of other healthcare interventions and settings. This work will help to establish the appropriateness of trust as additional construct in the TFA.

As the pilot trial was conducted in three months, during the busy winter period the concurrent work only included parents and therefore lacks insight into staff perspectives during pilot trial conduct.

For peer review only - http://bmjopen.bmj.com/site/about/guidelines.xhtml

This limitation was compensated for by the use of retrospective (1 week-1 month) mixed methods ensuring a larger sample, through the survey and depth of insights, through focus groups. Insight was gained into the views of 8 (2 interviews 6 questionnaires) out of 18 parents (44%) who had declined their child's continued participation in one or more aspect of the pilot RCT. In particular, the interviews with parents who declined consent and nursing staff who found the protocol challenging to follow provided valuable information to assist with refining the study process for a definitive RCT. However, it is unknown whether or not the predominantly positive views of the declining parents who took part in an interview or questionnaire were shared by other parents who declined the FEVER pilot RCT.

In summary challenges to delivering the proposed trial included staff and parent concerns about the acceptability of the proposed protocol. Pre-trial research, staff training and experience of pilot trial conduct augmented views, providing insight into how challenges may be overcome, such as changes to the inclusion criteria and delivery of site training. We present an adapted TFA to inform the design of future trial feasibility studies.

Acknowledgements

The authors would like to thank all the parents who shared their experiences with us; their contribution to the research is invaluable. The authors are grateful to all the staff at participating UK hospitals and charities/ support groups for their help with recruitment and the UK PICS Study Group (PICS-SG) for their contributions to this study. We would also like to thank Ms Clara Francis (CF) as co-investigator and patient partner.

Funding

This study was funded by the UK National Institute for Health Research Health Technology Assessment programme (15/44/01) and supported by the National Institute for Health Research Great Ormond Street Hospital Biomedical Research Centre. The trial sponsor was Great Ormond Street Hospital NHS Foundation Trust, Joint R&D Office GOSH/ICH, 30 Guilford Street, London WC1N 1EH, United Kingdom (email: Research.Governance@gosh.nhs.uk). The Clinical Trials Unit for the Fever study was the ICNARC CTU. The views expressed in this publication are those of the authors and not necessarily of the funder, the sponsor, the NHS, the NIHR, or the UK Department of Health.

Ethics approval and consent to participate

Ethical approval for the study was provided by the National Research Ethics Committee (NRES) (17/LO/1139). Management approvals were obtained from all study sites.

For peer review only

Data statement

The datasets generated during and/or analysed during the current study are not publicly available as consent was not sought for data sharing.

Competing interests' statement.

MJP is a member of the NIHR HTA Board. KMR is a member of the NIHR HS & DR Board.

Author contributions

Dr Elizabeth Deja conducted the mixed methods research, co-analysed the data, drafted the initial manuscript, reviewed and revised the manuscript.

Professor Mark J Peters is the chief investigator of the FEVER study, he conceived, designed and oversaw conduct of the FEVER study and critically reviewed the manuscript.

Mr Imran Khan was the study manager at the ICNARC CTU responsible for day to day FEVER study management and critically reviewed the manuscript.

Mr Paul R Mouncey is the head of research at ICNARC CTU, a co-applicant, involved in the design and coordination of the FEVER study, contributed to and reviewed the manuscript.

Mr Blaise Fenn and Mr Jason Watkins were study co-applicants, patient and parent representatives who contributed to the design and conduct of the FEVER study including mixed methods perspectives elements.

Dr Padmanabhan Ramnarayan co-applicant helped design and conduct the FEVER study.

Dr Rachel Agbeko, Dr Shane M Tibby, Professor Lyvonne N Tume were co-applicants, site principal investigators and critically reviewed the manuscript.

Dr Kentigern Thorburn site principal investigator and critically reviewed the manuscript.

Dr Kathryn M Rowan is the director at ICNARC CTU, study co-applicant involved in designing and overseeing the FEVER study.

Dr Kerry Woolfall was a co-applicant on the FEVER study, designed the mixed methods perspectives elements of the FEVER study, supervised the mixed methods research, co- analysed the data and reviewed and revised the manuscript.

Licence statement

I, the Submitting Author has the right to grant and does grant on behalf of all authors of the Work (as defined in the below author licence), an exclusive licence and/or a non-exclusive licence for contributions from authors who are: i) UK Crown employees; ii) where BMJ has agreed a CC-BY licence shall apply, and/or iii) in accordance with the terms applicable for US Federal Government officers or employees acting as part of their official duties; on a worldwide, perpetual, irrevocable, royalty-free basis to BMJ Publishing Group Ltd ("BMJ") its licensees and where the relevant Journal is co-owned by BMJ to the co-owners of the Journal, to publish the Work in BMJ Open and any other BMJ products and to exploit all rights, as set out in our licence.

For peer review only - http://bmjopen.bmj.com/site/about/guidelines.xhtml

REFRENCES

1. McDonald AM, Knight RC, Campbell MK, et al. What influences recruitment to randomised controlled trials? A review of trials funded by two UK funding agencies. Trials. 2006;7(9).
2. Bower P, Brueton V, Gamble C, et al. Interventions to improve recruitment and retention in clinical trials: a survey and workshop to assess current practice and future priorities. Trials. 2014;15(399).
3. Treweek S, Mitchell E, Pitkethly M, et al. Strategies to improve recruitment to randomised controlled trials. Cochrane Database Syst Re. 2010.
4. Caldwell P, Murphy S, Butow P, Craig J. Clinical trials in children The Lancet. 2004;364(9436):803-11.
5. Greenberg RG, Gamel B, Bloom D, et al. Parents' perceived obstacles to pediatric clinical trial participation: Findings from the clinical trials transformation initiative Contemporary Clinical Trials Communications. 2018.
6. Sammons HM, Starkey ES. Ethical issues of clinical trials in children. Paediatrics and child health 2011;22(2).
7. Field MJ, Behrman RE. Institute of Medicine (U.S.). Committee on Clinical Research Involving Children., Ethical Conduct of Clinical Research Involving Children. Washington, D.C.: National Academies Press; 2004.
8. Naka F, Strober B, Shahriari M. Clinical trials: Kids are not just little people. Clinics in Dermatology. 2017;35(6):583-93.
9. Caldwell PHY, Butow PN, Craig JC. Pediatricians' attitudes towards randomized controlled trials involving children. The Journal of Pediatrics 2002;141(6).
10. Caldwell P, Butow P, Craig J. Parents' attitudes to randomised controlled trials involving children. Journal of Pediatricts 2003;145:555–60.
11. Enpr-EMA. Preparedness of medicines' clinical trials in paediatrics. Amsterdam: European Medicines Agency; 2020.
12. Diepeveen S, Ling T, Suhrcke M, Roland M, Marteau TM. Public acceptability of government intervention to change health-related behaviours: a systematic review and narrative synthesis. BMC Public health 2013;13(756).
13. Sekhon M, Cartwright M, Francis JJ. Acceptability of healthcare interventions: an overview of reviews and development of a theoretical framework. BMC Health Services Research. 2017;17(88).
14. Brick T, Agbeko RS, Davies P, et al. Too hot to handle: a survey of attitudes towards fever of 462 pediatric intensive care unit staff. Eur J Pediatr 2016;176:423–7.
15. NICE. Fever in Under 5s: Assessment and Initial Management. In: guideline N, editor. London: National Institute for Health and Care Excellence; 2019. p. 1-36.
16. Proposal for a Regulation of the European Parliament and of the Council on Clinical trials on medicinal products for human use, and repealing Directive 2001/20/EC., (2013).
17. The Medicines for human use (Clinical trials) and Blood Safety and Quality (Amendment) Regulations., (2008).
18. Peters MJ, Woolfall K, Khan I, et al. Permissive versus restrictive temperature thresholds in critically ill children with fever and infection: a multicentre randomized clinical pilot trial. Critical Care. 2019;23(1):69.
19. Peters MJ, Woolfall K, Khan I, et al. Different temperature thresholds for antipyretic intervention in critically ill children with fever due to infection: the FEVER feasibility RCT. Health Technology Assessment 2019;23(5):1 - 148.
20. Woolfall K, O'Hara C, Deja E, et al. Parents' prioritised outcomes for trials investigating treatments for paediatric severe infection: a qualitative synthesis. Arch Dis Child. 2019;0:1-6.
21. Woolfall K, Frith L, Gamble C, et al. How parents and practitioners experience research without prior consent (deferred consent) for emergency research involving children with life threatening conditions: a mixed method study. BMJ Open. 2015;5.

For peer review only - http://bmjopen.bmj.com/site/about/guidelines.xhtml

22. Inwald DP, Canter R, Woolfall K, et al. Restricted fluid bolus volume in early septic shock: results of the Fluids in Shock pilot trial. Arch Dis Child. 2018.
23. Molyneux S, Njue M, Boga M, et al. The words will pass with the blowing wind': staff and parent views of the deferred consent process, with prior assent, used in an emergency fluids trial in two African hospitals. PLOS ONE 2013;8(8).
24. Inwald D, Canter R, Woolfall K, et al. Restricted fluid bolus versus current practice in children with septic shock: the FiSh feasibility study and pilot RCT. Health Technol Assess. 2018;22(51).
25. Saunder B, Sim J, Kingstone T, et al. Saturation in qualitative research: exploring its conceptualization and operationalization. Qual Quant. 2018;52(4).
26. Braun V, Clarke V. Using thematic analysis in psychology. Qualitative research in psychology. 2006;3(2):77-101.
27. Braun V, Clarke V. What can "thematic analysis''offer health and wellbeing researchers? International journal of qualitative studies on health and well-being. 2014;9:26152.
28. Gale NK, Heath G, Cameron E, Rashid S, Redwood S. Using the framework method for the analysis of qualitative data in multi-disciplinary health research. BMC Medical Research Methodology. 2013;13(117).
29. O'Cathain A, Thomas KJ, Drabble SJ, Rudolph A, Goode J, Hewison J. Maximising the value of combining qualitative research and randomised controlled trials in health research: the QUAlitative Research in Trials (QUART) study--a mixed methods study. Health Technol Assess. 2014;18(38):1-197, v-vi.
30. Canvin K, Jacoby A. Duty, desire or indifference? A qualitative study of patient decisions about recruitment to an epilepsy treatment trial. Trials. 2006;7(32).
31. Roper L, Sherratt FC, Young B, et al. Children's views on research without prior consent in emergency situations: a UK qualitative study. BMJ open 2018;8.
32. Waterfield T, Lyttle MD, Shields M, et al. Parents' and clinicians' views on conducting paediatric diagnostic test accuracy studies without prior informed consent: qualitative insight from the Petechiae in Children study (PiC). Archives of Disease in Childhood. 2019:317.
33. Woolfall K, Frith L, Dawson A, Gamble C, Lyttle MD, Young B. Fifteen-minute consultation: an evidence-based approach to research without prior consent (deferred consent) in neonatal and paediatric critical care trials. Archives of disease in childhood - Education and amp; practice edition. 2016;101(1):49-53.
34. Brierley J, Larcher V. Emergency research in children: options for ethical recruitment. Journal of Medical Ethics. 2011;37(7):429-32.
35. Hall MA, Dugan E, Zheng B, Mishra AK. Trust in Physicians and Medical Institutions:What Is It, Can It Be Measured,and Does It Matter? The Milbank Quarterly. 2001;79(4).

For peer review only

For peer review only - http://bmjopen.bmj.com/site/about/guidelines.xhtml

LEDGENDS

Figure 1: Sekhon, Cartwright and Francis (2017) Theoretical Framework of Acceptability

Figure 2: Fever Feasibility study design

Figure 3: Participant characteristics by time point

Table 1: Approach to qualitative data analysis

Table 2: Prospective Acceptability of a FEVER Pilot Trial mapped to Sekhon et al's acceptability framework (2017)

Table 3: Parent concordant acceptability of a FEVER Pilot Trial mapped to Sekhon et al's acceptability framework (2017)

Table 4: Respective Acceptability of a FEVER Pilot Trial mapped to Sekhon et als's acceptability framework (2017)

Figure 4: The adapted Theoretical framework of Acceptability

For peer review only

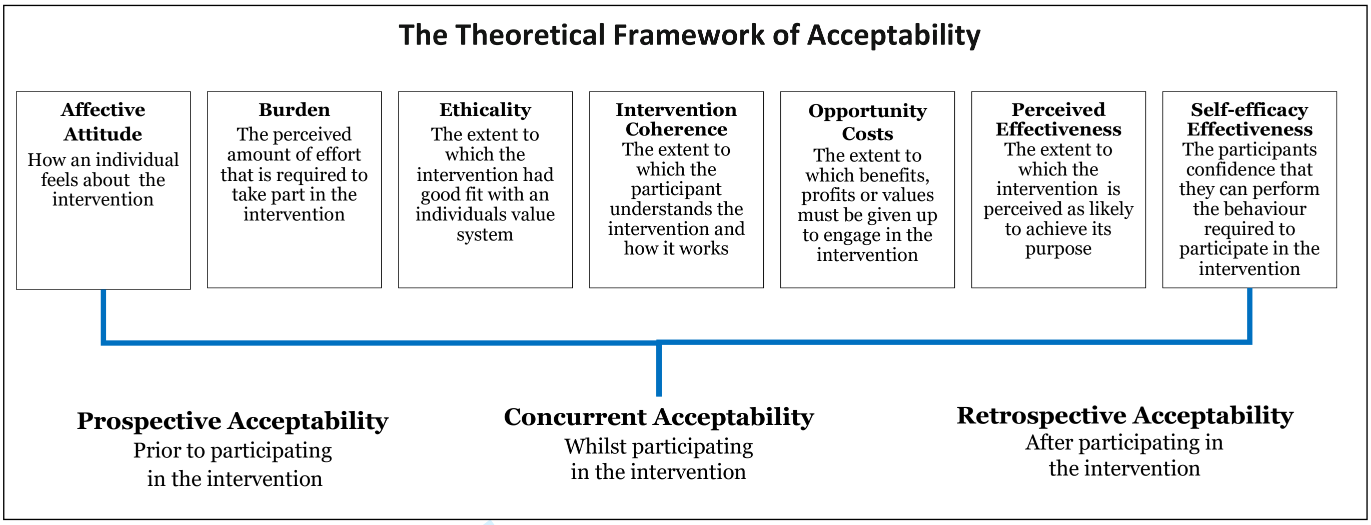

peer review only

Stage 1

Pre trial feasibility research including:

- Interviews with Parents with experience their child being admitted to an intensive care unit with a fever and suspected infection in the preceding 3 years
- Focus groups with clinicians (nurses and doctors) working the 4 PICUs planned to be included in the pilot
- Observational study of UK practice related to fever management*

Stage 2

- Pilot RCT in 4 hospitals comparing a permissive approach (treat as ≥40°C) with a restrictive approach (treat at ≥37.5°C) to fever management in children*
- Embedded survey to explore parent perspectives at the point of trial recruitment.

Stage 3

- Interviews with parents of children randomised to the pilot RCT approximately one month after hospital discharge.
- Survey and focus groups with staff involved in the pilot RCT at the end of trial recruitment.

*reported separately

Prospective: Pre pilot RCT

Parents

Interviews, n = 25
Mothers, n = 20, 80%, 4 bereaved
Fathers, n = 5, 20%, 2 bereaved

Practitioners

Focus groups, n = 56
Nurses, n = 45, 81%
Doctors, n = 11, 19%

⇩

Concordant: During pilot RCT

Parents

Questionnaires, n = 48 (from 47 families)
Mothers, n = 38, 79.2%/ Fathers, n = 10, 20.8%
Consented, n = 41/48, 85.4% / not consented, n = 6/48, 12.5% / n=1 missing

⇩

Prospective: Post pilot RCT

Parents

Interviews, n = 19/ 101, 18.8%
Mothers, n = 13/19, 68.4%
Fathers, n = 6/19, 31.6%
Consented, n = 17/19, 89.5%
Not consented, n = 2/19, 10.5%

Practitioners

Focus groups, n = 50, 51%
Questionnaire, n = 48, 49%
Total, n = 98
Nurses , n = 75, 76.5%
Doctors n = 14, 14.3
Other n = 8, 8.1%
Missing, n = 1, 1%

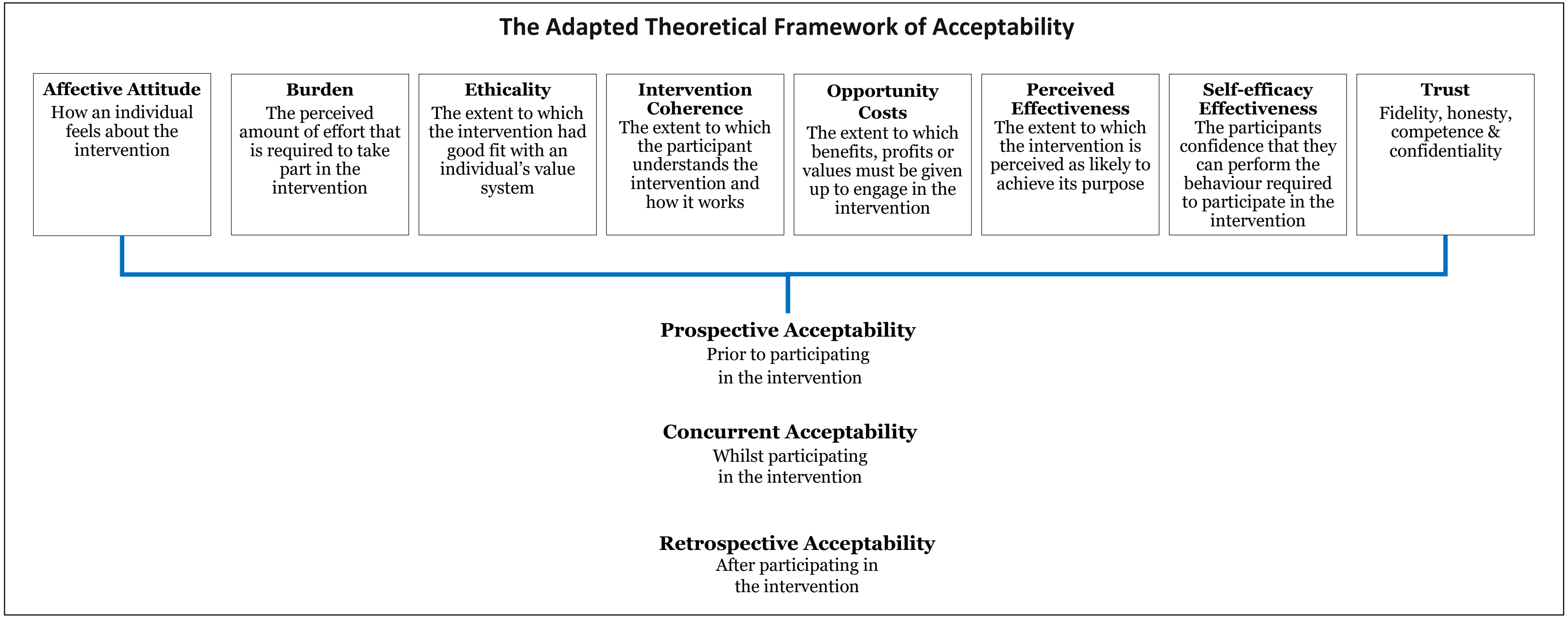
The Adapted Theoretical Framework of Acceptability
Affective Attitude
How an individual feels about the intervention
Burden
The perceived amount of effort that is required to take part in the intervention
Ethicality
The extent to which the intervention had good fit with an individual's value system
Intervention Coherence
The extent to which the participant understands the intervention and how it works
Opportunity Costs
The extent to which benefits, profits or values must be given up to engage in the intervention
Perceived Effectiveness
The extent to which the intervention is perceived as likely to achieve its purpose
Self-efficacy Effectiveness
The participants confidence that they can perform the behaviour required to participate in the intervention
Trust
Fidelity, honesty, competence & confidentiality
Prospective Acceptability
Prior to participating in the intervention
Concurrent Acceptability
Whilst participating in the intervention
Retrospective Acceptability
After participating in the intervention

COREQ (COnsolidated criteria for REporting Qualitative research) Checklist

A checklist of items that should be included in reports of qualitative research. You must report the page number in your manuscript where you consider each of the items listed in this checklist. If you have not included this information, either revise your manuscript accordingly before submitting or note N/A.

Topic	Item No.	Guide Questions/Description	Reported on Page No.
**Domain 1: Research team and reflexivity**			
Personal characteristics			
Interviewer/facilitator	1	Which author/s conducted the interview or focus group?	
Credentials	2	What were the researcher's credentials? E.g. PhD, MD	
Occupation	3	What was their occupation at the time of the study?	
Gender	4	Was the researcher male or female?	
Experience and training	5	What experience or training did the researcher have?	
Relationship with participants			
Relationship established	6	Was a relationship established prior to study commencement?	
Participant knowledge of the interviewer	7	What did the participants know about the researcher? e.g. personal goals, reasons for doing the research	
Interviewer characteristics	8	What characteristics were reported about the inter viewer/facilitator? e.g. Bias, assumptions, reasons and interests in the research topic	
**Domain 2: Study design**			
Theoretical framework			
Methodological orientation and Theory	9	What methodological orientation was stated to underpin the study? e.g. grounded theory, discourse analysis, ethnography, phenomenology, content analysis	
Participant selection			
Sampling	10	How were participants selected? e.g. purposive, convenience, consecutive, snowball	
Method of approach	11	How were participants approached? e.g. face-to-face, telephone, mail, email	
Sample size	12	How many participants were in the study?	
Non-participation	13	How many people refused to participate or dropped out? Reasons?	
Setting			
Setting of data collection	14	Where was the data collected? e.g. home, clinic, workplace	
Presence of non-participants	15	Was anyone else present besides the participants and researchers?	
Description of sample	16	What are the important characteristics of the sample? e.g. demographic data, date	
Data collection			
Interview guide	17	Were questions, prompts, guides provided by the authors? Was it pilot tested?	
Repeat interviews	18	Were repeat inter views carried out? If yes, how many?	
Audio/visual recording	19	Did the research use audio or visual recording to collect the data?	
Field notes	20	Were field notes made during and/or after the inter view or focus group?	
Duration	21	What was the duration of the inter views or focus group?	
Data saturation	22	Was data saturation discussed?	
Transcripts returned	23	Were transcripts returned to participants for comment and/or	

For peer review only

Topic	Item No.	Guide Questions/Description	Reported on Page No.
		correction?	
**Domain 3: analysis and findings**			
Data analysis			
Number of data coders	24	How many data coders coded the data?	
Description of the coding tree	25	Did authors provide a description of the coding tree?	
Derivation of themes	26	Were themes identified in advance or derived from the data?	
Software	27	What software, if applicable, was used to manage the data?	
Participant checking	28	Did participants provide feedback on the findings?	
Reporting			
Quotations presented	29	Were participant quotations presented to illustrate the themes/findings? Was each quotation identified? e.g. participant number	
Data and findings consistent	30	Was there consistency between the data presented and the findings?	
Clarity of major themes	31	Were major themes clearly presented in the findings?	
Clarity of minor themes	32	Is there a description of diverse cases or discussion of minor themes?	

Developed from: Tong A, Sainsbury P, Craig J. Consolidated criteria for reporting qualitative research (COREQ): a 32-item checklist for interviews and focus groups. *International Journal for Quality in Health Care*. 2007. Volume 19, Number 6: pp. 349 – 357

Once you have completed this checklist, please save a copy and upload it as part of your submission. DO NOT include this checklist as part of the main manuscript document. It must be uploaded as a separate file.

For peer review only
